# Genomic epidemiology of human candidaemia isolates in a tertiary hospital

**DOI:** 10.1099/mgen.0.001047

**Published:** 2023-07-13

**Authors:** Ka Lip Chew, Rosemini Achik, Nurul Hudaa Osman, Sophie Octavia, Jeanette W. P. Teo

**Affiliations:** ^1^​ Department of Laboratory Medicine, National University Hospital, Singapore, Singapore; ^2^​ Environmental Health Institute, National Environment Agency, Singapore, Singapore

**Keywords:** Antimicrobial resistance, Candidaemia, Whole-genome-sequencing, Genomic epidemiology, Antifungal resistance

## Abstract

Invasive candida infections are significant infections that may occur in vulnerable patients with high rates of mortality or morbidity. Drug-resistance rates also appear to be on the rise which further complicate treatment options and outcomes. The aims of this study were to describe the prevalence, molecular epidemiology, and genetic features of *Candida* bloodstream isolates in a hospital setting. The resistance mechanisms towards the two most commonly administered antifungals, fluconazole and anidulafungin, were determined. Blood culture isolates between 1 January 2018 and 30 June 2021 positive for *Candida* spp. were included. Susceptibility testing was performed using Etest. Whole-genome-sequencing was performed using Illumina NovaSeq with bioinformatics analysis performed. A total of 203 isolates were sequenced: 56 *C*. *glabrata,* 53 *C*. *tropicalis*, 44 *C*. *albicans,* 36 *C*. *parapsilosis* complex (consisting of *C. parapsilosis, C. orthopsilosis,* and *C. metapsilosis*), six *C*. *krusei,* five *C*. *dubliniensis*, and three *C*. *auris*. A single cluster of azole-resistant *C. tropicalis,* and four clusters of *C. parapsilosis* isolates were observed, suggesting possible transmission occurring over several years. We found 11.3%, and 52.7 % of *C. tropicalis* and *C. parapsilosis*, respectively, clustered with other isolates, suggesting exogenous sources may play a significant role of transmission, particularly for *C. parapsilosis*. The clusters spanned over several years suggesting the possibility of environmental reservoirs contributing to the spread. Limited clonality was seen for *C. albicans*. Several sequence types appeared to be dominant for *C. glabrata,* however the SNP differences varied widely, indicating absence of sustained transmission.

## Data Summary

All genome data for this study have been deposited in GenBank. The genome raw reads have been deposited in the Short Read Archive as SRR19696069 with the BioProject No. PRJNA848263. The authors confirm all supporting data and protocols have been provided within the article or through supplementary data files.

**Impact Statement**

Infections with *Candida* spp. are traditionally thought to be endogenous in nature (arising from one’s own flora). Our data demonstrate differences in diversity between *Candida* spp*.,* with clustering of non-albicans *Candida* spp. over several years. Whilst limited clustering is seen for some species, >50 % of *C. parapsilosis* clustered with other isolates. This suggests the possibility of exogenous sources of infection and occult transmission may occur in healthcare settings. The sporadic occurrence of clustered isolates over varying locations and at different times suggest the possibility of periodic breaks in infection control, and possible environmental reservoirs of infection.

## Introduction

Invasive candidiasis are significant infections that may occur in patients, particularly those who have critical illness or are immunocompromised. Typical risk factors for candidaemia include ICU admission, total parenteral nutrition use, malignancy, and neutropaenia. High mortality rates are reported even with antifungal therapy [1]. Currently, first line therapy for patients are echinocandins [2]. Fluconazole may be considered as a step-down oral therapy in some patients where susceptible [2, 3]. Antifungal resistance, however, is problematic. Increasing acquired resistance to fluconazole is seen over time, particularly in non-albicans *Candida* spp. [4, 5]. In such situations, echinocandins are used for the entire treatment duration, which is difficult as it requires intravenous administration, and is costlier than fluconazole. As echinocandins are first-line-therapy, the presence of acquired resistance, although rare, may result in a delay in effective antifungal therapy and contribute to worse outcomes [4].

Historically, the four most commonly isolated pathogenic *Candida* spp. are *Candida albicans, Candida tropicalis, Candida parapsilosis,* and *Candida glabrata*. In addition, *Candida auris* has joined the fray causing a global outbreak in ICUs, with the capacity to acquire resistance against azoles, echinocandins, as well as polyenes [6–8]. This has brought to the attention of the medical community, evidence of a fungal species causing clonal outbreaks and required tightening of infection control practices. However, clonal outbreaks of resistant *Candida* spp. are not limited to *C. auris* and has been reported in *C. parapsilosis* and *C. tropicalis* [9, 10]. This also challenges the notion that invasive infections with *Candida* spp. are usually caused by an individual’s own normal flora.

The aims of this study were to describe the prevalence, molecular epidemiology, and genetic features of *Candida* bloodstream isolates in a hospital setting. We investigated possible transmission patterns of not just drug-resistant but also drug-susceptible *Candida* spp.*,* providing insight into how candidaemia infections may have been acquired.

## Methods

### Clinical setting

**Fig. 1.**
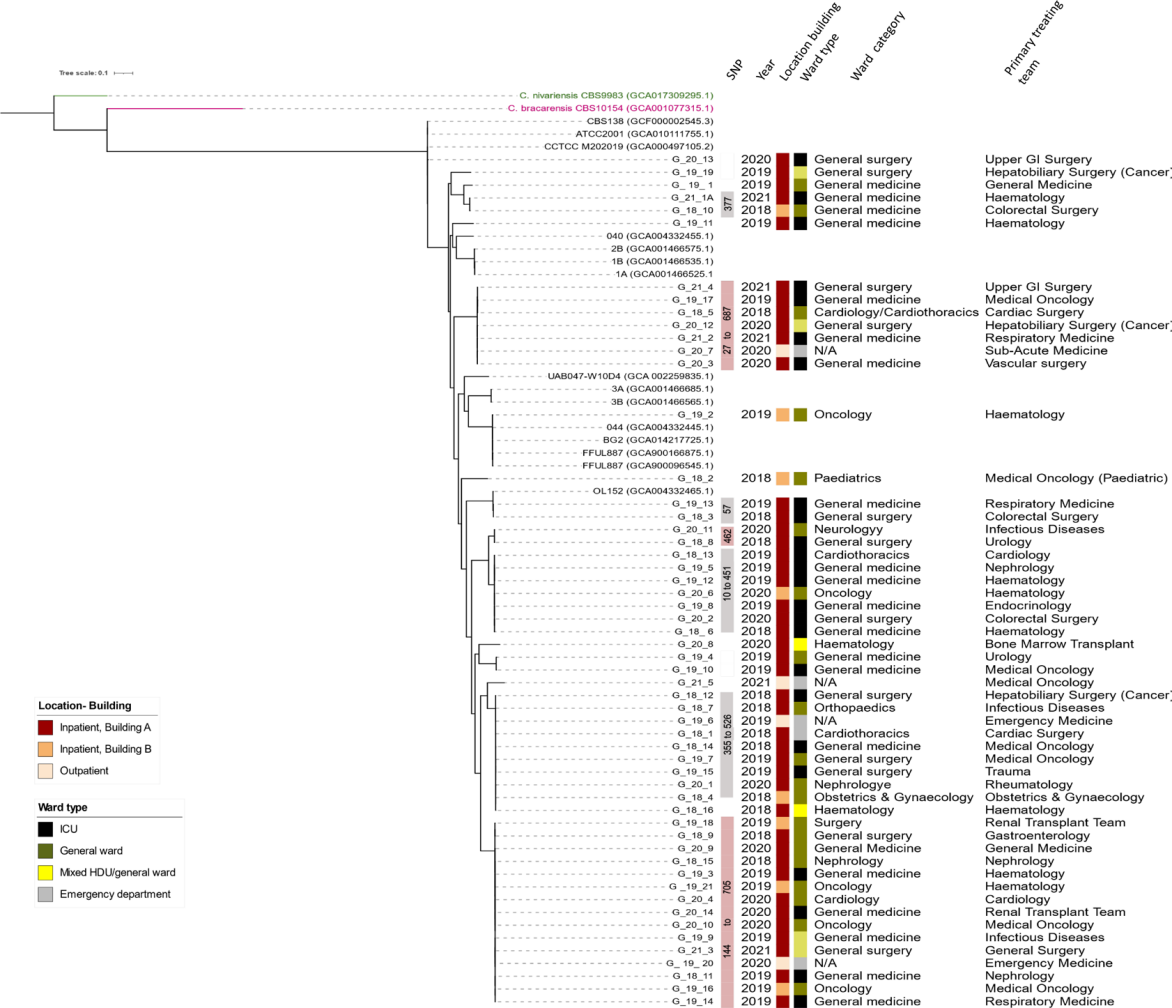
Phylogeny of *Candida glabrata*. The trees were constructed from SKA (Split Kmer Analysis). MLST sequence types (ST) is indicated. GenBank accession numbers of assemblies used as comparators are written in parenthesis. All isolates sequenced in this study have the prefix G_ (for *C. glabrata*).

**Fig. 2.**
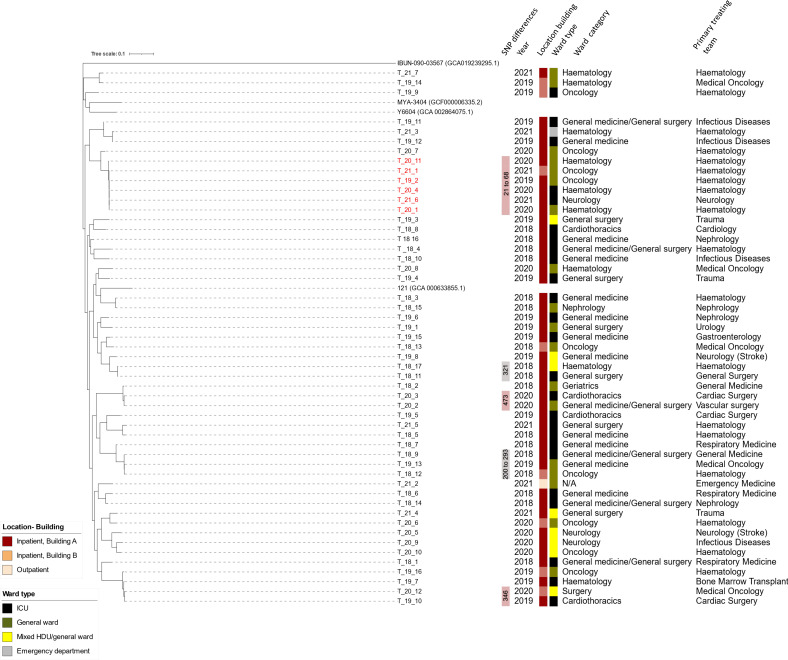
Phylogeny of *Candida tropicalis*. Azole-resistant cluster of isolates are coloured in red. The trees were constructed from SKA (Split Kmer Analysis). GenBank accession numbers of assemblies used as comparators are written in parenthesis. All isolates sequenced in this study have the prefix T_ (for *C. tropicalis*).

**Fig. 3.**
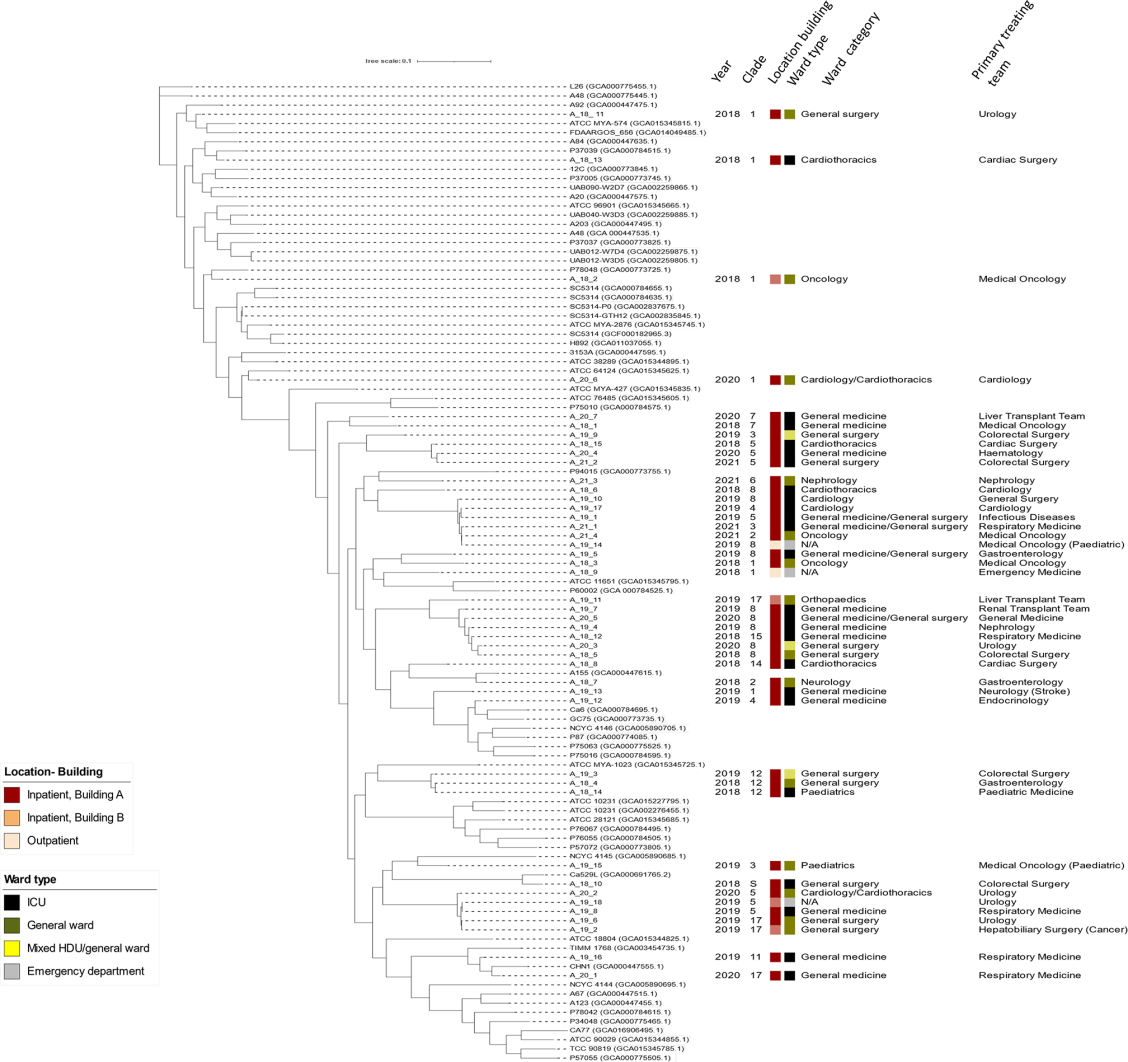
Phylogeny of *Candida albicans*. The trees were constructed from SKA (Split Kmer Analysis). GenBank accession numbers of assemblies used as comparators are written in parenthesis. All isolates sequenced in this study have the prefix A_ (for *C. albicans*).

**Fig. 4.**
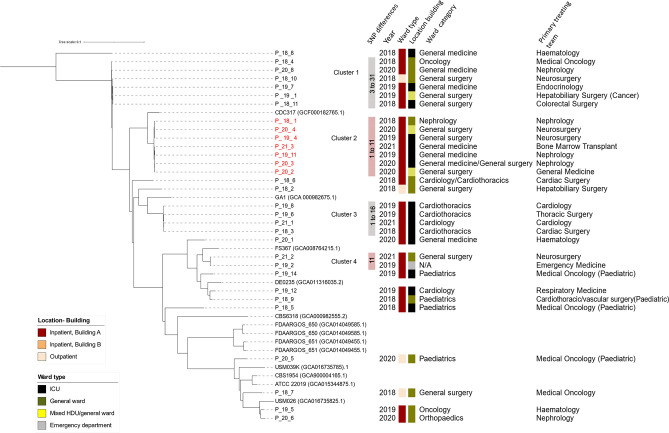
Phylogeny of *Candida parapsilosis*. Four clusters of *C. parapsilosis* are demonstrated, including a azole-resistant cluster with isolates coloured in red. The trees were constructed from SKA (Split Kmer Analysis). GenBank accession numbers of assemblies used as comparators are written in parenthesis. All isolates sequenced in this study have the prefix P_ (for *C. parapsilosis*).

The study was performed at a 1200-bed tertiary referral hospital. The range of services offered here are broad including adult, paediatric, obstetrics and gynaecology services, medicine and surgery across a wide range of specialties including solid organ and haematology oncology, solid organ transplant (kidney, liver, and pancreas), and stem cell transplant, as well as intensive care services (including neonatal). Inpatients services are provided across two separate blocks with wards. The wards were broadly categorized in this study into intensive care units (ICU), high dependency units (HDU), general ward, and emergency department. Individual wards are usually assigned particular patient types and these are broadly categorized as ward category (e.g. General medicine, General surgery) and associated primary treatment teams (e.g. Medical Oncology, Infectious diseases) (Figs 1–4.).

### *Candida* isolates

Blood culture isolates between 1 January 2018 and 30 June 2021 positive for *Candida* spp. were included in this study. Blood cultures were incubated using the BacT/Alert Virtuo (Biomérieux, Marcy-l'Étoile, France) system, upon positive signal, an aliquot of blood was subcultured onto routine culture media (blood agar, chocolate agar, and McConkey agar) and Sabouraud dextrose agar when fungi were seen on Gram-stain. Identification of isolates was performed using MALDI-TOF (Bruker MALDI Biotyper, Billerica, Massachusetts, US).

### Antifungal susceptibility testing

Susceptibility testing for fluconazole, anidulafungin, and amphotericin B was performed using Etest (Biomérieux, Marcy-l'Étoile, France). The minimum-inhibitory-concentration (MIC) results were interpreted based on EUCAST breakpoints. Where breakpoints were not available, non-species-specific breakpoints were applied for fluconazole (with the exception of *C. krusei* due to the presence of intrinsic resistance mechanisms), and a susceptible breakpoint of ≤1 mg l^−1^ was applied for amphotericin B as this was the common breakpoint used for *Candida* spp. Only non-duplicate isolates (first isolate from a patient) were included in describing the susceptibility profiles.

### Whole-genome-sequencing, whole-genome alignment and SNP calling

Illumina NovaSeq was used to generate 150 bp paired-end reads. The raw reads were then assembled using Shovill v1.0.4 (https://github.com/tseemann/shovill) coupled with SPAdes v. 3.14.0 [11]. SKA v1.0 (Split kmer analysis) [12] was used to generate split kmer files based on raw reads (ska fastq, default parameters) and reference-free whole-genome alignments were produced (ska align). SNP distance was calculated using snp-dists (version 0.7.0) (https://github.com/tseemann/snp-dists). FastTree [13] with a general time reversible (GTR+CAT) model was used to construct the phylogenetic trees with 1000 bootstrap replicates. iTOL [14] was then used to visualise the phylogenetic trees. Snippy v2 tool (https://github.com/tseemann/snippy) was used for SNP variant calling of drug-resistance associated loci. Snippy uses BWA MEM [15] for read-alignment and Freebayes [16] for variant calling. The default parameters for Snippy were used. Annotated GenBank sequences for *ERG3*, *ERG11*, *FKS* genes, efflux-transporters and transcriptional factors that mediate drug resistance (Table S1, available in the online version of this article) were used as a reference for Snippy to determine the presence of non-synonymous mutations which may confer drug resistance phenotype. Copy number variation in *RTA3*, *ERG3* and *ERG11* genes of *C. parapsilosis* genomes was determined using pipeCoverage (https://github.com/MrOlm/pipeCoverage).

## Multilocus sequence typing (MLST)

For pathogenic *Candida* species, formal multilocus sequence typing (MLST) schemes have been published for *C. albicans*, *C. dubliniensis*, *C. tropicalis*, *C. glabrata*, and *C. krusei* [17]. The MLST schemes comprise six to eight housekeeping genes and was determined using the PubMLST database (https://pubmlst.org/organisms/). *C. parapsilosis* does not currently have an MLST scheme. For *C. albicans* clade assignment, the MLST alleles were concatenated for cluster analysis and the clade defined as previously described [18]. The genome raw reads have been deposited in the Short Read Archive as SRR19696069 with the BioProject No. PRJNA848263. The individual accession numbers are provided in Table S2. All isolates sequenced in this study have a naming prefix of G_ (for *C. glabrata*), T_ (for *C. tropicalis*), A_ (for *C. albicans*) and P_ (for *C. parapsilosis*).

## Results and discussion

### Species distribution of *Candida* blood stream isolates

Between 1 January 2018 and 30 June 2021, there were 203 non-duplicate *Candida* spp. isolated from blood cultures. Seven different *Candida s*pp. were isolated and *C. glabrata* was the most common cause of bloodstream infection (28.2 %), followed by *C. tropicalis* (25.3 %) and *C. albicans* (21 %) (Table 1). The detailed species breakdown is as follows: 56 *C*. *glabrata* complex (renamed as *Nakaseomyces glabrata* complex and includes *Nakaseomyces nivariensis* and *Nakaseomyces bracarensis*) [19], 53 *C*. *tropicalis*, 44 *C*. *albicans,* 36 *C*. *parapsilosis* complex (consisting of *C. parapsilosis, C. orthopsilosis,* and *C. metapsilosis*), six *C*. *krusei* (renamed as *Pichia kudriavzevii*) [19], five *C*. *dubliniensis*, and three *C*. *auris* [19].

**Table 1.** Susceptibility profiles of candidaemia isolates to fluconazole and anidulafungin

*Candida* spp.	Year (*N*)	Fluconazole			Anidulafungin	
		S	I	R	S	R
***C. glabrata*** (*n*=56)	2018 (16)	n/a	87.5 % (14/16)	12.5 % (2/16)	100 % (16/16)	0
	2019 (21)	n/a	95.2 % (20/21)	4.8 % (1/21)	90.5 % (19/21)	9.5 % (2/21)
	2020 (14)	n/a	78.6 % (11/14)	21.4 % (3/14)	100 % (14/14)	0
	2021 (5)	n/a	100 % (5/5)	0	100 % (5/5)	0
	2018 (17)	94.1 % (16/17)	5.9 % (1)	0	82.4 % (14/17)	17.6 % (3/17)
***C. tropicalis***	2019 (17)	88.2 % (15/17)	0	11.8 % (2/17)	100 % (17/17)	0
(*n*=53)	2020 (12)	66.7 % (8/12)	0	33.3 % (4/12)	100 % (12/12)	0
	2021 (7)	57.1 % (4/7)	0	42.9 % (3/7)	100 % (7/7)	0
						
	2018 (15)	100 % (15/15)	0	0	100 % (15/15)	0
***C. albicans***	2019 (18)	100 % (18/18)	0	0	100 % (18/18)	0
(*n*=44)	2020 (7)	100 % (7/7)	0	0	100 % (7/7)	0
	2021 (4)	100 % (4/4)	0	0	100 % (4/4)	0
	2018 (11)	90.9 % (10/11)	0	9.1 % (1/11)	100 % (11/11)	0
***C. parapsilosis***	2019 (14)	78.6 % (11/14)	0	21.4 % (3/14)	100 % (14/14)	0
(*n*=36)	2020 (8)	50 % (4/8)	12.5 % (1/8)	37.5 % (3/8)	87.5 % (7/8)	12.5 % (1/8)
	2021 (3)	33.3 % (1/3)	0	66.7 % (2/3)	100 % (3/3)	0
	2018 (0)	n/a	n/a	n/a	0	0
***C. krusei***	2019 (3)	n/a	n/a	n/a	3 (100 %)	0
(*n*=6)	2020 (3)	n/a	n/a	n/a	3 (100 %)	0
	2021 (0)	n/a	n/a	n/a	0	0
	2018 (0)	0	0	0	n/a	n/a
***C. auris***	2019 (0)	0	0	0	n/a	n/a
(*n*=3)	2020 (2)	0	0	2 (100 %)	n/a	n/a
	2021 (1)	0	0	1 (100 %)	n/a	n/a
	2018 (1)	1 (100 %)	0	0	n/a	n/a
***C. dubliniensis***	2019 (0)	0	0	0	n/a	n/a
(*n*=5)	2020 (2)	2 (100 %)	0	0	n/a	n/a
	2021 (2)	2 (100 %)	0	0	n/a	n/a

Based on EUCAST susceptibility testing definitions (https://www.eucast.org/fileadmin/src/media/PDFs/EUCAST_files/EUCAST_Presentations/2018/EUCAST_-_Intermediate_category_-_information_for_all.pdf).

I, Susceptible, increased exposure; n, number of isolates; N/A, Not applicable as there are currently no established clinical breakpoints for the species; R, Resistant; S, Susceptible, standard dosing reigmen.

Of the 36 *C. parapsilosis*, 31 isolates were *C. parapsilosis sensu stricto*, four were *C. orthopsilosis* and one isolate was *C. metapsilosis*. A similar prevalence and distribution of the three species was also observed in other hospitals where *C. parapsilosis sensu stricto* was consistently the most dominant species and *C. metapsilosis* the least common [20, 21].

*C. nivariensis* and *C. bracarensis*, rare pathogens which are closely related to *C. glabrata sensu stricto* and also known to cause invasive disease, were not detected in our cohort. This was similar to global findings that both *C. nivariensis* and *C. bracarensis* were extremely uncommon (0.2 % prevalence amongst *C. glabrata* complex isolates) [22].

The species distribution parallels other observational studies. A 12 month, laboratory-based surveillance of candidaemia at 25 hospitals from China, Hong Kong, India, Singapore, Taiwan and Thailand showed that *C. albicans* was most frequently isolated (41.3%) while *C. tropicalis* was the leading non-albicans species [23]. Differences in species variation are due to a myriad of factors including geography, hospital settings, patient populations and standards of prophylaxis and empiric therapy.

The demographics and primary treatment teams for included patients are summarized based on *Candida* spp*.* in Tables S3-S6. These are further stratified based on whether isolates formed clusters versus non-clustered isolates for *C. tropicalis* and *C. parapsilosis,* and sequence type for *C. glabrata*. The average age was 61 years with more male (*n*=124) than female (*n*=79) patients with no clear predilection seen for any particular pathogen. Overall, the treating teams with the most number of candidaemia cases were the haematology team (16.3 % of all cases), followed by medical oncology (9.9 %), and nephrology (7.4 %). This is consistent with known risk factors for candidaemia.

### Phenotypic antifungal susceptibility profiles

The overall susceptibility results for fluconazole and anidulafungin are summarised in Table 1. Fluconazole susceptibility rates decreased progressively from 2018 especially for *C. parapsilosis* complex and *C. tropicalis* isolates. *C. albicans* was found to be completely susceptible to both azole and echinocandins (Table 1). Susceptibility data from Asia-Pacific region [23] showed lowered fluconazole susceptibility in non-albicans species particularly *C. tropicalis, C. glabrata,* and *C. parapsilosis*, similar to the trends observed here.

Overall, echinocandin resistance rates was low. In *C. tropicalis, C. glabrata* and *C. parapsilosis*, between 2.8–5.5 % of the isolates were echinocandin resistant (Table 1). Previous susceptibility data from the Asia-Pacific region (years 2013–2015) [23] showed complete susceptibility suggesting an upward trend of echinocandin resistance. Anidulafungin resistance during the first episode of candidaemia was seen in only two *C. glabrata* isolates, one *C. parapsilosis,* and three *C. tropicalis* (Table 1). One case of acquired anidulafungin resistance in *C. glabrata* occurred whilst on treatment within 14 days from the index blood culture. With the exception of a single isolate of *C. auris* in 2021, all isolates were susceptible to amphotericin B (data not shown).

### Molecular determinants of antifungal resistance

### Genetic resistance to azoles

**Fig. 5.**
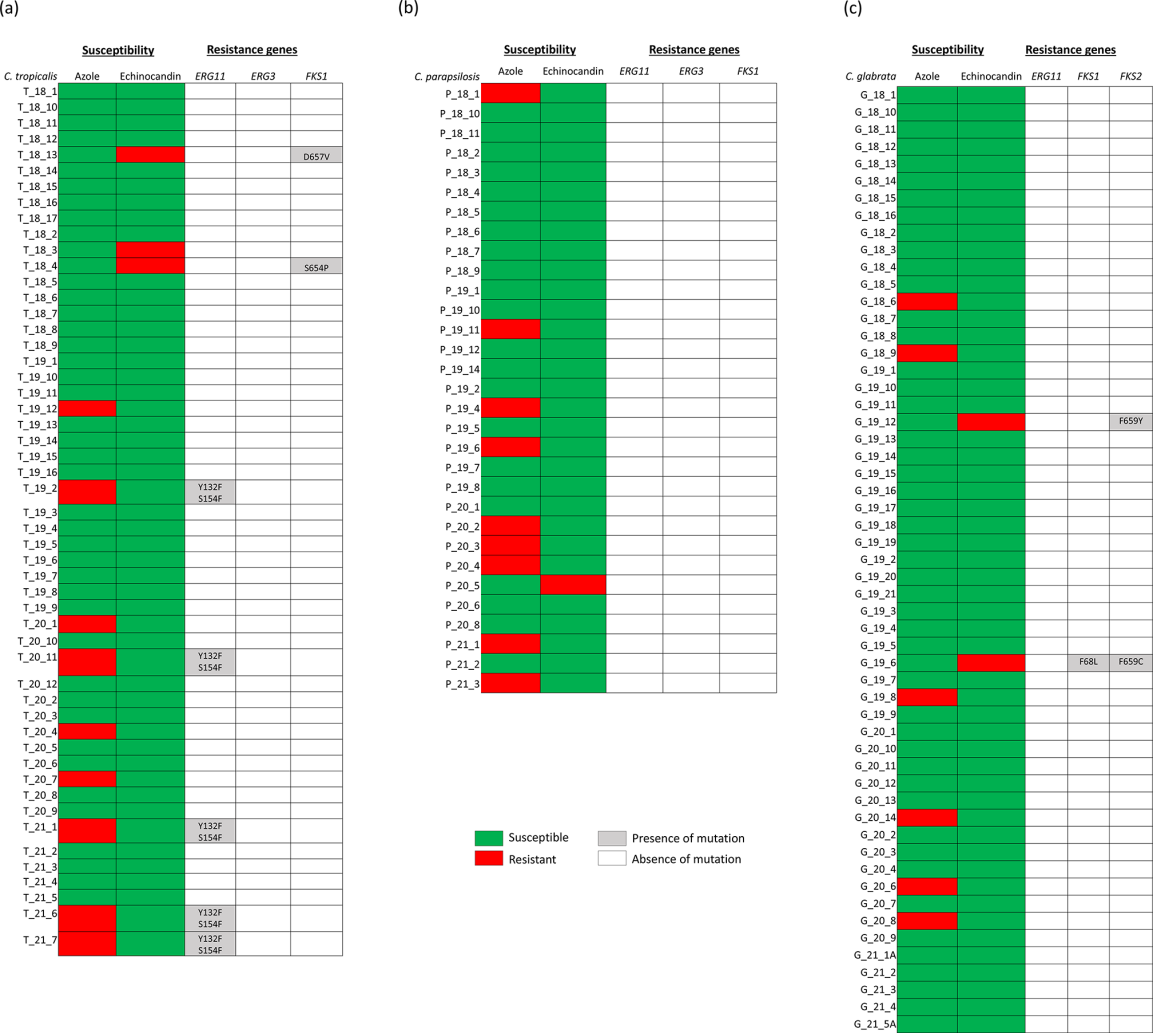
Heatmap distribution of mutations in *FKS* and *ERG Candida* genes and phenotypic antifungal susceptibility towards azole and echinocandin. The accession numbers for the loci are listed in supplementary data.

Five out of nine azole resistant *C. tropicalis* isolates carried Y132F and S154F double mutation in ERG11p which mediates high-level azole resistance [24] (Fig. 5a). The other four had no mutation detected suggesting a wild-type *ERG11.* Overexpression of *MDR* transporters and *CDR* efflux pumps (Table S1) could increase resistance to azole antifungal drugs [25]. However, no missense mutations were detected in the implicated drug-loci of fluconazole-resistant *C. tropicalis*.

Y132F substitution in ERG11p is correlated with azole resistance in *C. parapsilosis* [26]. However, none of the resistant isolates (9/36) carried this mutation (Fig. 5b). No polymorphisms in the other *ERG* loci (*ERG2*, *ERG3*, *ERG4*, *ERG6* and *ERG25*) were correlated with azole resistance. Gain-of-function (GOF) mutations were not observed in transcriptional factors *UPC2*, *NDT80*, *MRR1* and *TAC1*. This suggests that there may be a novel mechanism of azole resistance perhaps yet undetected in the clinical isolates.

Gene copy number gains have been implicated in fluconazole resistance. The gene *RTA3*, encoding a lipid translocase, has been implicated in antifungal resistance, when found in multiple copies [27]. Analysis of our *C. parapsilosis* genomes for *RTA3*, *ERG3* and *ERG11* did not reveal any duplications.

*C. glabrata* has an intrinsic resistance to fluconazole, with no obvious *ERG11* polymorphism correlated to resistance [28] (Fig. 5c). GOF variants of transcription factors PDR1p behave as hyperactive inducers of transcription and elicit high-level fluconazole resistance via overexpression of target genes such as the ABC transporter-encoding gene *CDR1*, *CDR2* and *SNQ2* [29, 30]. *C. glabrata PDR1* has a high allelic diversity. This was demonstrated in a study looking at 122 clinical *C. glabrata* isolates. At least 12 different alleles recovered only from azole-susceptible isolates containing combinations of eight different mutations and 58 different alleles specific for azole-resistant isolates with 58 distinct mutations [29]. Four single amino acid substitutions in PDR1p (W297S, F575L, P822L and P927L) have been shown in separate studies to increase the expression of *CDR1* thus contributing to azole resistance of clinical isolates [29], we observed shared polymorphisms in both our azole-susceptible and azole-resistant isolates (Table S7). GOF mutations that were previously described were not seen in our isolates. However, we did observe unique PDR1p mutations R282G, L291Q, L732S in isolates with high fluconazole MICs (>256 mg l^−1^). A previously observed missense mutation L935M was seen in an isolate with azole MIC of 16 mg l^−1^ (Table S7). This L935M mutation is located on the central regulatory domain [30] and future expression studies are needed to determine their role in target gene activation.

All three *C. auris* were azole-resistant and harboured the Y132F mutation in ERG11p, which is characteristic of clade I isolates [31]. *C. krusei* has natural intrinsic resistance to fluconazole [32]. All *C. dubliniensis* isolates were azole susceptible and correspondingly we did not observe *ERG* amino acid polymorphisms.

In summary, mutations in ERG11p were the most predominant mechanism of azole resistance in our *C. tropicalis* and *C. auris* isolates. The role of mutations in genes encoding transcription factors or efflux pumps appear to be minimal.

### Resistance to echinocandins

While resistance to azoles can be due to mutations in several genes which code for different functional categories including drug transporters, target site mutations, and target overexpression, the echinocandin resistance mechanism is more restricted [33]. Echinocandin resistance resulting in clinical failures is conferred by a limited number of amino acid substitutions in the conserved ‘hot-spot’ (HS) regions of the *FKS1* and *FKS2* subunits of the glucan synthase.

Both echinocandin resistant *C. glabrata* isolates carried a F659 mutation (F659Y, F659C) in Fks2p. The F659 position has been established to be important for decreased echinocandin susceptibility [34] (Fig. 5c).

Of the three anidulafungin resistant *C. tropicalis* isolates, two isolates had mutations in Fks1p while the remaining isolate had none (Fig. 5a). S654P is a known resistance conferring mutation [33] and was harboured in the isolate with MIC 0.5 mg l^−1^. Whilst there is no evidence regarding Fks1p D657V, it was found in the other isolate with MIC 0.19 mg l^−1^ (resistant) and could potentially represent a novel drug mutation.

*C. parapsilosis* has an intrinsically reduced susceptibility to echinocandins resulting from a naturally occurring P660A substitution in Fks1p. This mutation leads to a characteristic 2-log decrease in echinocandin sensitivity [35]. A single resistant isolate with MIC 8 mg l^−1^ did not display any mutations in the *FKS* genes (Fig. 5b). This was consistent with previous findings that isolates with an appreciable range of echinocandin MIC values did not harbour amino acid substitutions in HS1 and/or HS2 of Fks1p [36]. For *C. krusei* and *C. dubliniensis,* no FKS mutations were detected.

About 7 % of clade I *C. auris* are resistant to echinocandins [6]. Of the three *C. auris* isolates, two had anidulafungin MICs of 0.125 mg l^−1^, and the other had an MIC of 1.0 mg l^−1^. Fks1p M690I mutation was detected in the isolate with anidulafungin MIC of 1 mg l^−1^, whilst the other two isolates had wild-type *FKS1.* Fks1p M690I mutation has not been described but could represent a novel resistance conferring mutation.

We focussed on investigating well-established mechanisms of resistance including drug target alteration of *ERG* and *FKS* genes and gain-of-function mutations in transcription factors and its efflux transporters. However, the genomes of drug-resistant strains after antifungal treatment often exhibit large-scale changes, such as loss of heterozygosity (LOH), copy-number variation (CNV) [37] including short segmental CNV, and whole chromosome aneuploidy. This has been well-demonstrated for *C. albicans*. We did not look at those global gene arrangements here as that analysis would be better suited with long-read sequencing.

### Clonal spread of azole-resistant *C. glabrata*, *C. tropicalis* and *C. parapsilosis*

#### Candida glabrata

For *C. glabrata sensu stricto*, the most prevalent sequence types (STs) for *C. glabrata* were: ST7, ST55, and ST59 (Tables 2 and S8). ST7 is the major genotype in bloodstream isolates seen in studies performed in South Korea, and China [38–40]. ST59 have also identified in bloodstream infections albeit at a much lower frequency than ST7 [41]. It has also been shown that ST7 was associated with an azole-resistant phenotype [38] although this was not observed here (Fig. 1). The primary treating teams are summarized in Table S6, stratified by the four most common STs seen. Three of seven (42.9%) ST59 isolates were treated by the haematology team, two of which were cultured from the same ward. However, all three occurred in different years (at least 9 months apart). In addition, the SNP differences between isolates within the same ST also varied widely. The most closely related isolates were ten SNPs apart (G_19_5 and G_19_12; ST 59). These isolates were cultured from the same ICU, although 4 months apart. The primary treating team for these two patients were the Haematology and Nephrology teams. While it remains possible that there was clonal spread of *C. glabrata,* this does not appear to be a dominant source of infection.

**Table 2.** Distribution of sequence types and clades across the different *Candida* spp

	Sequence type	No. of isolates (%)
*C. glabrata* (*n*=56)	7	15 (26.8)
	55	10 (17.9)
	59	7 (12.5)
	83	6 (10.7)
	8	2 (3.6)
	26	2 (3.6)
	24*	2 (3.6)
	46	2 (3.6)
	Singletons†	10 (17.8)
*C. tropicalis* (*n*=53)	New/unassigned	51 (96.2)
	Singletons	2 (3.8)
*C. albicans* (*n*=44)	New/unassigned	44 (100)
*C. krusei* (*n*=6)	New/unassigned	6 (100)
*C. parapsilosis* (*n*=36)	Clade 4‡	25
	Clade 5	6
	Clade 2	4
	Clade 3	1

*The closest assigned sequence type

†Singletons here refer to unique sequence types

‡Clades were assigned according to the phylogeny described by Bergin *et al.* [57]

#### Candida tropicalis

Population genomics study showed that *C. tropicalis* genomes could be highly genetically variable [42] with heterozygosity levels ranging from two to six variants per kb for most isolates. We note fairly extensive genetic variations between genomes (Fig. 2) where the majority (51/53, 96.2%) of the *C. tropicalis* isolates could not be assigned to known DSTs (Tables 2 and S8). Other studies have also shown extensive genetic variation reflected in the diversity of DSTs.

The *C. tropicalis* isolates in this study were divergent, with an exception of a cluster of six azole isolates (Fig. 2). This *C. tropicalis* cluster was associated with the haematology-oncology patients between 2019–2021. Five of six (83.3 %) isolates from the *C. tropicalis* cluster was isolated from haematology patients who were admitted to haematology-oncology wards (Fig. 2). Of these five patients, four were from the same ward, which suggests the possibility of direct transmission. The last patient was admitted in a separate building. This cluster of isolates spanned over 3 years (2019–2021).

#### Candida albicans

For *C. albicans*, all of their diploid sequence types (DST) were unique and previously unassigned (Table 2 and S8). Similarly, in an Italian study, 18 different DSTs were obtained from 21 *C. albicans* isolates and 72 % of these DSTs could not be assigned to any of the existing *C. albicans* genotypes in the MLST database [9].

*C. albicans* is known to exhibit a high degree of genetic diversity, notably with a high frequency of heterozygosity occurring along the genome [43]. At the population level, *C. albicans* belong to 18 well-defined clades with associated epidemiological characteristics, such as the site of infection, geographical distribution, and antifungal drug susceptibility [44].

Globally, the five most populous *C. albicans* clades were 1, 2, 3, 4, and 11 [18]. In our study of candidaemia isolates, the three most common clades were clade eight (9/44, 20.5 %), clade one isolates (8/44, 18.1 %) and clade five isolates (8/44, 18.1 %). Four (9.1 %) clade 17 isolates were detected (Fig. 3). Clade 17 (28.3 %) and clade one were the most and second most common in blood stream isolates in Thailand [45]. In Taiwan, bloodstream isolates belonged mostly to clades 1, 4, 3, and 17 [46].

Overall, our genotyping data had parallel observations from different geographical regions such as Kuwait [47], Germany [48], and Taiwan [46] where *C. albicans* isolates were largely heterogeneous, with no evidence of intrahospital transmission or correlation to antifungal resistance.

#### *Candida parapsilosis* complex

The population structure of *C. parapsilosis* is more ‘restricted’ as compared to *C. albicans*. This is consistent with data that *C. parapsilosis* itself is highly clonal, lacking sequence diversity, with low rates of heterozygosity at 1 SNP per 15 553 bases [26].

*C. parapsilosis* clustering was observed in 52.7 % of isolates (19/36), with <31 SNPs intra-cluster (Fig. 4). These were separated into four clusters with varying azole-resistance. The majority of resistant isolates clustered together (Cluster 2). For *C. parapsilosis* Cluster 3, there appears to be an association with location with three isolates being cultured from the same ward, between 2018–2019. All four patients had primary treating teams from cardiology and cardiothoracic surgery. For *C. parapsilosis* cluster 2, three of seven (42.9 %) of cases were patients whose primary treating team was Nephrology, although all three patients were in different locations at the time of culture, with all three cultures from different years (2018, 2019, and 2020). The other clusters do not appear to have a clear association location and patient population. Clustered *C. parapsilosis* isolates spanned all the years of collection (2018–2021) (Fig. 4). Persistence of *C. parapsilosis* clusters has been demonstrated in other studies [49]. Guinea *et al.* showed clonal persistence of *C. parapsilosis* spanning 4 years with genotype dominance. The authors also suggested that *C. parapsilosis* from the hands of healthy people and healthcare workers may contribute to the dominant persisting *C. parapsilosis* genotypes observed. Azole-resistant *C. parapsilosis* isolates appeared to have lower genetic diversity compared to the susceptible isolates and were more likely to cause clonal invasive infections [50].

Our data indicates clustering of both azole-resistant and azole-susceptible isolates (Fig. 4). Current literature also supports evidence for long-term (>2 years) environmental persistence of azole-resistant *C. parapsilosis* [10].

#### Phylogeny of *C. krusei, C. dubliniensis, C. auris* – the uncommon species

All of the six *C. krusei* isolates had different MLST allelic profiles (Tables 2 and S8) suggesting non-clonality amongst these isolates. Although an important pathogenic fungal species, the population genetic parameters of clinical *C. krusei* are largely unknown. A high rate of heterozygous SNP variants was observed in a clinical isolate *C. krusei* 81-B-5 [51]. It had an average rate of 1 SNP every 340 positions, higher than the rate reported in many *C. albicans* isolates [51]. Whether this variability extends to other clinical isolates and the effect on phylogeny remains to be studied.

None of the four *C. dubliniensis* isolates were genetically related and were characterized by the absence of clustering when compared to other publicly available genomes (Fig. S1).

Four major geographic clades of *C. auris* are recognised: South Asian (clade I), East Asian (clade II), South African (clade III), and South American (clade IV). *C. auris* was first detected in Singapore in 2012. Studies on the local genomic epidemiology of isolates have been limited but thus far the majority of the isolates belong to the clade I [52]. This was also the clade that all of our three isolates belonged to. SNP differences of fewer than 12 SNPs between patients are likely to represent recent transmission based on Chow *et al.* [53]. Here, the *C. auris* isolates had >30 SNPs between them and in comparison to isolates isolated in earlier years from another tertiary hospital, the SNP differences were large (>100 SNPs).

#### Phylogenetic relationships suggest overall lack of patient-to-patient transmissions

The emergence of multidrug-resistant *C. auris* which has repeatedly caused global outbreaks [54] raised the concern about fungal species as an infection control threat. Our data demonstrate that clonal spread of other *Candida* spp*.* can also occur. Although the overall population structure of *C. glabrata*, *C. tropicalis, C. parapsilosis, C. albicans,* and *C. krusei* was made up of diverse clones and not dominated by a single clone, several clusters of isolates were noted (Figs 1–4).

Clustering of isolates occurred most commonly with *C. parapsilosis,* with 19 of 36 (52.7 %) isolates. These were separated between four clusters with varying azole-resistance (Fig. 4). This was followed by *C. tropicalis with* six of 53 (11.3 %) clustered in a single azole-resistant *C. tropicalis* cluster predominantly involving haematology-oncology patients in haematology-oncology wards (21–68 SNPs) (Fig. 2). Two clusters of *C. glabrata* were also seen (five of 56 isolates, 8.9 %). There were no overlaps in the primary treating team for patients and often cases were located in different wards and were months to years apart. This suggests that patient-to-patient spread may play a less significant role however transmission between patients without clinical infection cannot be excluded. The sporadic nature (spread out over years and location) may also indicate intermittent breaks in infection control procedures and act as a signal to remind healthcare workers to maintain vigilance in daily care of patients. The interspersed nature of these isolates also suggests the possibility of these isolates being part of a lineage that has successfully adapted in hospital/healthcare environments. Clonal outbreaks of other *Candida* spp. has been reported, particularly isolates with acquired azole-resistance [9, 10]. This may be due to publication bias in that investigations are more likely to be triggered for isolates with drug-resistance.

*Candida* spp*.* have been demonstrated to survive on inanimate surfaces, particularly if there is moisture [55]. *C. albicans* appears to be least viable on inanimate surfaces, which correlates with our findings that they are most heterozygous with no evidence of *C. albicans* transmission between different patients. Conversely, *C. parapsilosis* survives well on inanimate surfaces, which supports its potential as a persistent nosocomial pathogen [56].

## Conclusions

In summary, we report the genomic epidemiology of candidaemia isolates, demonstrating a single cluster of isolates in azole-resistant *C. tropicalis,* and multiple clusters of *C. parapsilosis*. Azole-susceptibilities of *C. parapsilosis* varied between and within the clustered isolates. Clustering amongst other *Candida* spp*.* (with the exception of *C. auris*) was not evident. This may be due to endogenous infections being a more common source of some *Candida* spp*.* while exogenous (environmental sources) being more common for *C. tropicalis* and *C. parapsilosis.* Environmental testing was not performed for this study but would be of interest for future research directions to identify reservoirs that may contribute to nosocomial candidaemia infections. Other factors that were not investigated include antifungal usage and patient-specific risk factors.

Future investigations should investigate these factors in parallel in order to delineate relative risk that each factor contributes. This may identify potential interventions that can reduce overall candidaemia rates in hospital and particularly transmission of drug-resistant isolates.

## Supplementary Data

Supplementary material 1

## References

[1] Reboli AC, Rotstein C, Pappas PG, Chapman SW, Kett DH (2007). Anidulafungin versus fluconazole for invasive candidiasis. N Engl J Med.

[2] Pappas PG, Kauffman CA, Andes DR, Clancy CJ, Marr KA (2016). Clinical practice guideline for the management of Candidiasis: 2016 update by the infectious diseases society of America. Clin Infect Dis.

[3] Vazquez J, Reboli AC, Pappas PG, Patterson TF, Reinhardt J (2014). Evaluation of an early step-down strategy from intravenous anidulafungin to oral azole therapy for the treatment of candidemia and other forms of invasive candidiasis: results from an open-label trial. BMC Infect Dis.

[4] Chew KL, Octavia S, Lin RTP, Yan GZ, Teo JWP (2019). Delay in effective therapy in anidulafungin-resistant Candida tropicalis fungaemia: potential for rapid prediction of antifungal resistance with whole-genome-sequencing. J Glob Antimicrob Resist.

[5] Chew KL, Cheng JWS, Jureen R, Lin RTP, Teo JWP (2017). ERG11 mutations are associated with high-level azole resistance in clinical Candida tropicalis isolates, a Singapore study. Mycoscience.

[6] Chowdhary A, Prakash A, Sharma C, Kordalewska M, Kumar A (2018). A multicentre study of antifungal susceptibility patterns among 350 *Candida auris* isolates (2009-17) in India: role of the ERG11 and FKS1 genes in azole and echinocandin resistance. J Antimicrob Chemother.

[7] Escandón P, Chow NA, Caceres DH, Gade L, Berkow EL (2019). Molecular epidemiology of *Candida auris* in Colombia reveals a highly related, countrywide colonization with regional patterns in amphotericin B resistance. Clin Infect Dis.

[8] Lockhart SR, Etienne KA, Vallabhaneni S, Farooqi J, Chowdhary A (2017). Simultaneous emergence of multidrug-resistant *Candida auris* on 3 continents confirmed by whole-genome sequencing and epidemiological analyses. Clin Infect Dis.

[9] Scordino F, Giuffrè L, Barberi G, Marino Merlo F, Orlando MG (2018). Multilocus sequence typing reveals a new cluster of closely related *Candida tropicalis* genotypes in Italian patients with neurological disorders. Front Microbiol.

[10] Thomaz DY, de Almeida JN, Lima GME, Nunes M de O, Camargo CH (2018). An azole-resistant *Candida parapsilosis* outbreak: clonal persistence in the intensive care unit of a Brazilian teaching hospital. Front Microbiol.

[11] Bankevich A, Nurk S, Antipov D, Gurevich AA, Dvorkin M (2012). SPAdes: a new genome assembly algorithm and its applications to single-cell sequencing. J Comput Biol.

[12] Harris SR (2018). SKA: Split Kmer Analysis toolkit for bacterial genomic epidemiology. Genomics.

[13] Price MN, Dehal PS, Arkin AP (2009). FastTree: computing large minimum evolution trees with profiles instead of a distance matrix. Mol Biol Evol.

[14] Letunic I, Bork P (2016). Interactive tree of life (iTOL) v3: an online tool for the display and annotation of phylogenetic and other trees. Nucleic Acids Res.

[15] Li H, Durbin R (2009). Fast and accurate short read alignment with Burrows–Wheeler transform. Bioinformatics.

[16] Garrison E, Marth G (2012). Haplotype-based variant detection from short-read sequencing. arXiv preprint arXiv, 1207.3907.

[17] Odds FC, Jacobsen MD (2008). Multilocus sequence typing of pathogenic *Candida* species. Eukaryot Cell.

[18] Odds FC, Bougnoux M-E, Shaw DJ, Bain JM, Davidson AD (2007). Molecular phylogenetics of *Candida albicans*. Eukaryot Cell.

[19] Borman AM, Johnson EM, Kraft CS (2021). Name changes for fungi of medical importance, 2018 to 2019. J Clin Microbiol.

[20] Chen YC, Lin YH, Chen KW, Lii J, Teng HJ (2010). Molecular epidemiology and antifungal susceptibility of Candida parapsilosis sensu stricto, Candida orthopsilosis, and Candida metapsilosis in Taiwan. Diagn Microbiol Infect Dis.

[21] Miranda-Zapico I, Eraso E, Hernández-Almaraz JL, López-Soria LM, Carrillo-Muñoz AJ (2011). Prevalence and antifungal susceptibility patterns of new cryptic species inside the species complexes Candida parapsilosis and Candida glabrata among blood isolates from a Spanish tertiary hospital. J Antimicrob Chemother.

[22] Lockhart SR, Messer SA, Gherna M, Bishop JA, Merz WG (2009). Identification of Candida nivariensis and Candida bracarensis in a large global collection of *Candida glabrata* isolates: comparison to the literature. J Clin Microbiol.

[23] Tan TY, Hsu LY, Alejandria MM, Chaiwarith R, Chinniah T (2016). Antifungal susceptibility of invasive *Candida* bloodstream isolates from the Asia-Pacific region. Med Mycol.

[24] Jiang C, Dong D, Yu B, Cai G, Wang X (2013). Mechanisms of azole resistance in 52 clinical isolates of *Candida tropicalis* in China. J Antimicrob Chemother.

[25] Zuza-Alves DL, Silva-Rocha WP, Chaves GM (2017). An update on *Candida tropicalis* based on basic and clinical approaches. Front Microbiol.

[26] Tóth R, Nosek J, Mora-Montes HM, Gabaldon T, Bliss JM (2019). *Candida parapsilosis*: from genes to the bedside. Clin Microbiol Rev.

[27] Whaley SG, Tsao S, Weber S, Zhang Q, Barker KS (2016). The RTA3 gene, encoding a putative lipid translocase, influences the susceptibility of *Candida albicans* to fluconazole. Antimicrob Agents Chemother.

[28] Hassan Y, Chew SY, Than LTL (2021). *Candida glabrata*: pathogenicity and resistance mechanisms for adaptation and survival. JoF.

[29] Ferrari S, Ischer F, Calabrese D, Posteraro B, Sanguinetti M (2009). Gain of function mutations in CgPDR1 of *Candida glabrata* not only mediate antifungal resistance but also enhance virulence. PLoS Pathog.

[30] Simonicova L, Moye-Rowley WS (2020). Functional information from clinically-derived drug resistant forms of the *Candida glabrata* Pdr1 transcription factor. PLOS Genet.

[31] Chaabane F, Graf A, Jequier L, Coste AT (2019). Review on antifungal resistance mechanisms in the emerging pathogen *Candida auris*. Front Microbiol.

[32] Guinea J, Sánchez-Somolinos M, Cuevas O, Peláez T, Bouza E (2006). Fluconazole resistance mechanisms in *Candida krusei*: the contribution of efflux-pumps. Med Mycol.

[33] Pfaller MA (2012). Antifungal drug resistance: mechanisms, epidemiology, and consequences for treatment. Am J Med.

[34] Costa-de-Oliveira S, Marcos Miranda I, Silva RM, Pinto E Silva A, Rocha R (2011). FKS2 mutations associated with decreased echinocandin susceptibility of *Candida glabrata* following anidulafungin therapy. Antimicrob Agents Chemother.

[35] Garcia-Effron G, Katiyar SK, Park S, Edlind TD, Perlin DS (2008). A naturally occurring proline-to-alanine amino acid change in Fks1p in Candida parapsilosis, Candida orthopsilosis, and Candida metapsilosis accounts for reduced echinocandin susceptibility. Antimicrob Agents Chemother.

[36] Martí-Carrizosa M, Sánchez-Reus F, March F, Cantón E, Coll P (2015). Implication of *Candida parapsilosis* FKS1 and FKS2 mutations in reduced echinocandin susceptibility. Antimicrob Agents Chemother.

[37] Ford CB, Funt JM, Abbey D, Issi L, Guiducci C (2015). The evolution of drug resistance in clinical isolates of *Candida albicans*. Elife.

[38] Boonsilp S, Homkaew A, Phumisantiphong U, Nutalai D, Wongsuk T (2021). Species distribution, antifungal susceptibility, and molecular epidemiology of *Candida* species causing candidemia in a tertiary care hospital in Bangkok, Thailand. J Fungi.

[39] Byun SA, Won EJ, Kim MN, Lee WG, Lee K (2018). Multilocus Sequence Typing (MLST) genotypes of *Candida glabrata* bloodstream isolates in Korea: association with antifungal resistance, mutations in mismatch repair gene (Msh2), and clinical outcomes. Front Microbiol.

[40] Xiao M, Fan X, Hou X, Chen SC, Wang H (2018). Candida tropicalis and *Candida glabrata* isolates with delineation of their resistance mechanisms]]>. IDR.

[41] Biswas C, Marcelino VR, Van Hal S, Halliday C, Martinez E (2018). Whole genome sequencing of Australian *Candida glabrata* isolates reveals genetic diversity and novel sequence types. Front Microbiol.

[42] O’Brien CE, Oliveira-Pacheco J, Ó Cinnéide E, Haase MAB, Hittinger CT (2021). Population genomics of the pathogenic yeast *Candida tropicalis* identifies hybrid isolates in environmental samples. PLoS Pathog.

[43] Muñoz M, Wintaco LM, Muñoz SA, Ramírez JD (2019). Dissecting the heterogeneous population genetic structure of *Candida albicans*: limitations and constraints of the multilocus sequence typing scheme. Front Microbiol.

[44] Zhu Y, Fang C, Shi Y, Shan Y, Liu X (2022). *Candida albicans* multilocus sequence typing clade I contributes to the clinical phenotype of vulvovaginal candidiasis patients. Front Med.

[45] Pham LTT, Pharkjaksu S, Chongtrakool P, Suwannakarn K, Ngamskulrungroj P (2019). A predominance of clade 17 *Candida albicans* isolated from hemocultures in a tertiary care Hospital in Thailand. Front Microbiol.

[46] Wang SH, Shen M, Lin HC, Sun PL, Lo HJ (2015). Molecular epidemiology of invasive *Candida albicans* at a tertiary hospital in northern Taiwan from 2003 to 2011. Med Mycol.

[47] Al-Obaid K, Asadzadeh M, Ahmad S, Khan Z (2017). Population structure and molecular genetic characterization of clinical Candida tropicalis isolates from a tertiary-care hospital in Kuwait reveal infections with unique strains. PLoS One.

[48] Huyke J, Martin R, Walther G, Weber M, Kaerger K (2015). *Candida albicans* bloodstream isolates in a German university hospital are genetically heterogenous and susceptible to commonly used antifungals. Int J Med Microbiol.

[49] Guinea J, Mezquita S, Gómez A, Padilla B, Zamora E (2021). Whole genome sequencing confirms *Candida albicans* and *Candida parapsilosis* microsatellite sporadic and persistent clones causing outbreaks of candidemia in neonates. Med Mycol.

[50] Fekkar A, Blaize M, Bouglé A, Normand AC, Raoelina A (2021). Hospital outbreak of fluconazole-resistant *Candida parapsilosis*: arguments for clonal transmission and long-term persistence. Antimicrob Agents Chemother.

[51] Cuomo CA, Shea T, Yang B, Rao R, Forche A (2017). Whole genome sequence of the heterozygous clinical isolate *Candida krusei* 81-B-5. G3.

[52] Tan YE, Teo JQ-M, Rahman NBA, Ng OT, Kalisvar M (2019). Candida auris in Singapore: genomic epidemiology, antifungal drug resistance, and identification using the updated 8.01 VITEK2 system. Int J Antimicrob Agents.

[53] Chow NA, de Groot T, Badali H, Abastabar M, Chiller TM (2019). Potential fifth clade of *Candida auris*, Iran, 2018. Emerg Infect Dis.

[54] Forsberg K, Woodworth K, Walters M, Berkow EL, Jackson B (2019). *Candida auris*: the recent emergence of a multidrug-resistant fungal pathogen. Med Mycol.

[55] Piedrahita CT, Cadnum JL, Jencson AL, Shaikh AA, Ghannoum MA (2017). Environmental surfaces in healthcare facilities are a potential source for transmission of *Candida auris* and other *Candida* species. Infect Control Hosp Epidemiol.

[56] Welsh RM, Bentz ML, Shams A, Houston H, Lyons A (2017). Survival, persistence, and isolation of the emerging multidrug-resistant pathogenic yeast *Candida auris* on a plastic health care surface. J Clin Microbiol.

[57] Bergin SA, Zhao F, Ryan AP, Müller CA, Nieduszynski CA (2022). Systematic analysis of copy number variations in the pathogenic yeast *Candida parapsilosis* identifies a gene amplification in *RTA3* that is associated with drug resistance. mBio.

