# Supplementary material for: Genomic epidemiology of human candidaemia isolates in a tertiary hospital
Source: Microb Genom. 2023 Jul 13;9(7):mgen001047. doi: 10.1099/mgen.0.001047 (PMC10438824; doi:10.1099/mgen.0.001047)
Supplement: Supplementary material 1 [file mgen-9-1047-s001.pdf]

Supplementary Table 1. List of genes associated with resistance in *Candida* spp.

Species	Locus tag or Accession number	Gene	Product
Candida albicans	CAALFM_C500660CA	ERG11	Lanosterol 14-alpha-demethylase
	CAALFM_C104770CA	ERG3	C-5 sterol desaturase
	CAALFM_C102420CA	FKS1	1,3-beta-glucan synthase
C. glabrata	LS398136	ERG2	C-8 sterol isomerase
	GVI51_F01551	ERG3	C-5 sterol desaturase
	CAGL0A00429g	ERG4	Putative C24 sterol reductase
	NC006031	ERG6	C-24 sterol methyltransferase
	MK458539	ERG11	Lanosterol 14-alpha-demethylase
	XM_449421	CDR1 putative	Multidrug resistance protein
	XM_444792.1	PDR1	Transcription factor
	XM_447461.1	SNQ2	Multidrug resistance protein
	XP_446088	PDH1	Pleiotropic Drug Resistance (PDR) Family protein
	HQ845283	FKS1	1,3-beta-glucan synthase
	GVI51_K03883	FKS2	1,3-beta-glucan synthase
	CAGL0D00242g	CNE1	Role in protein folding, ubiquitin-dependent ERAD pathway and integral component of endoplasmic reticulum membrane localization
		EPA13	Sub-telomerically encoded lectin-like adhesin
C. parapsilosis	MK924381	ERG11	Lanosterol 14-alpha-demethylase
	KT277771	ERG3	C-5 sterol desaturase
	CPAR2_201490	ERG2	Predicted C-8 sterol isomerase
	CPAR2_502980	ERG4	Sterol reductase
	CPAR2_405010	ERG6	Sterol 24-C-methyltransferase
	CPAR2_801410	ERG25	C-4 methylsterol oxidase
	CPAR2_405290	CDR1	Multidrug resistance protein
	EU221325	FKS1	1,3-beta-glucan synthase
	CPAR2_807270	*MRR1*	Regulator of MDR1 transcription

	XM_036807730	TAC1 putative	Similar to *C. albicans* TAC1, transcriptional activator of drug-responsive genes including CDR1 and CDR2
	CPAR2_301760	MDR1 putative	Plasma membrane multidrug efflux pump
	CPAR2_207280	UPC2 putative	Similar to *C. albicans* UPC2, transcriptional regulator of ergosterol biosynthetic genes and sterol uptake
	CPAR2_213640	NDT80	Similar to *C. albicans* NDT80, activator of CDR1 induction by antifungal drugs
C. tropicalis	KC542319	ERG3	C-5 sterol desaturase
	MK906190	ERG11	Lanosterol 14-alpha-demethylase
	XM_002551139	ERG1	Squalene monooxygenase
	XM_002550035	ERG2	Sterol isomerase
	XM_002550136	ERG 3	C-5 sterol desaturase
	XM_002547516	ERG24	similar to C-14 sterol reductase
	NW_003020038	CDR1	Multidrug resistance protein
	XM_002547994	CDR2	Multidrug resistance protein
	XM_002545459	CDR3	ABC transporter
	KP938799	MDR1	Multidrug resistance protein
	XM_002548069	MDR1	Benomyl/methotrexate resistance protein
	XM_002547926, CPAR2_807270	MRR1 putative	Regulator of MDR1 transcription
	XM_002550963	TAC1 putative	Transcriptional activator of drug-responsive genes
	EU676168	FKS1	1,3-beta-glucan synthase
C. dubliniensis	Cd36_04520	ERG3	C-5 sterol desaturase
	Cd36_50660	ERG11	Lanosterol 14-alpha-demethylase
C. auris	CJI96_0000987	ERG1	Squalene epoxidase
	CJI96_0003733	ERG2	C-8 sterol isomerase
	CJI96_0002270	ERG3	C-5 sterol desaturase
	CJI96_0000691	ERG4	Similar to sterol C-24 reductase
	CJI96_0004850	ERG5	C-22 sterol desaturase

	CJI96_0004127	ERG6	Delta(24)-sterol C-methyltransferase
	CJI96_0002263	ERG10	Acetyl-CoA acetyltransferase
	CJI96_0003797	ERG7	2,3-epoxysqualene-lanosterol cyclase
	CJI96_0001197	ERG11	Lanosterol 14-alpha-demethylase
	CJI96_0001251	ERG8	Putative phosphomevalonate kinase
	CJI96_0004705	ERG12	Mevalonate kinase
	CJI96_0004564	ERG9	Putative farnesyl-diphosphate farnesyl transferase
	CJI96_0004651	ERG13	3-hydroxy-3-methylglutaryl coenzyme A synthase
	CJI96_0001659	ERG20	Putative farnesyl pyrophosphate synthetase
	CJI96_0000856	ERG24	C-14 sterol reductase
	CJI96_0001089	ERG25	C-4 methyl sterol oxidase
	CJI96_0003179	ERG26	C-3 sterol dehydrogenase
	CJI96_0005271	ERG27	3-Keto sterol reductase
	CJI96_0001597	CDR1	Multidrug resistance protein
	CJI96_0003612	CDR22	Multidrug transporter
	CJI96_0001761	Hmg1	HMG-CoA reductase
	CJI96_0005139	MDR1	MDR/MFS multidrug efflux pump
	CJI96_0001761	MRR1	Hydroxymethylglutaryl-CoA_reductase
	CJI96_0003110	MVD	Mevalonate diphosphate decarboxylase
	CJI96_0002973	SNQ	Transporter
	CJI96_0004336	TAC2	Transcriptional activator
	CJI96_0000996	UPC2	Transcription factor

Supplementary Table 2: Bioproject accession numbers (Short Read Archive SRR19696069, BioProject No. PRJNA848263)

GenBank Accession	Isolate name	Organism
SAMN28991151	A_18_1	Candida albicans
SAMN28991152	A_18_10	Candida albicans
SAMN28991153	A_18_11	Candida albicans
SAMN28991154	A_18_12	Candida albicans
SAMN28991155	A_18_13	Candida albicans
SAMN28991156	A_18_14	Candida albicans
SAMN28991157	A_18_15	Candida albicans
SAMN28991158	A_18_2	Candida albicans
SAMN28991159	A_18_3	Candida albicans
SAMN28991160	A_18_4	Candida albicans
SAMN28991161	A_18_5	Candida albicans
SAMN28991162	A_18_6	Candida albicans
SAMN28991163	A_18_7	Candida albicans
SAMN28991164	A_18_8	Candida albicans
SAMN28991165	A_18_9	Candida albicans
SAMN28991166	A_19_1	Candida albicans
SAMN28991167	A_19_10	Candida albicans
SAMN28991168	A_19_11	Candida albicans
SAMN28991169	A_19_12	Candida albicans
SAMN28991170	A_19_13	Candida albicans
SAMN28991171	A_19_14	Candida albicans
SAMN28991172	A_19_15	Candida albicans
SAMN28991173	A_19_16	Candida albicans
SAMN28991174	A_19_17	Candida albicans
SAMN28991175	A_19_18	Candida albicans
SAMN28991176	A_19_2	Candida albicans
SAMN28991177	A_19_3	Candida albicans
SAMN28991178	A_19_4	Candida albicans
SAMN28991179	A_19_5	Candida albicans
SAMN28991180	A_19_6	Candida albicans
SAMN28991181	A_19_7	Candida albicans
SAMN28991182	A_19_8	Candida albicans
SAMN28991183	A_19_9	Candida albicans
SAMN28991184	A_20_1	Candida albicans
SAMN28991185	A_20_2	Candida albicans
SAMN28991186	A_20_3	Candida albicans
SAMN28991187	A_20_4	Candida albicans
SAMN28991188	A_20_5	Candida albicans
SAMN28991189	A_20_6	Candida albicans
SAMN28991190	A_20_7	Candida albicans
SAMN28991191	A_21_1	Candida albicans

SAMN28991192	A_21_2	Candida albicans
SAMN28991193	A_21_3	Candida albicans
SAMN28991194	A_21_4	Candida albicans
SAMN28991195	Au_20_1	Candida auris
SAMN28991196	Au_20_2	Candida auris
SAMN28991197	Au_21_1	Candida auris
SAMN28991198	D_18_1	Candida dubliniensis
SAMN28991199	D_20_1	Candida dubliniensis
SAMN28991200	D_20_2	Candida dubliniensis
SAMN28991201	D_21_1	Candida dubliniensis
SAMN28991202	G_18_1	Candida glabrata complex
SAMN28991203	G_18_10	Candida glabrata complex
SAMN28991204	G_18_11	Candida glabrata complex
SAMN28991205	G_18_12	Candida glabrata complex
SAMN28991206	G_18_13	Candida glabrata complex
SAMN28991207	G_18_14	Candida glabrata complex
SAMN28991208	G_18_15	Candida glabrata complex
SAMN28991209	G_18_16	Candida glabrata complex
SAMN28991210	G_18_2	Candida glabrata complex
SAMN28991211	G_18_3	Candida glabrata complex
SAMN28991212	G_18_4	Candida glabrata complex
SAMN28991213	G_18_5	Candida glabrata complex
SAMN28991214	G_18_6	Candida glabrata complex
SAMN28991215	G_18_7	Candida glabrata complex
SAMN28991216	G_18_8	Candida glabrata complex
SAMN28991217	G_18_9	Candida glabrata complex
SAMN28991218	G_19_1	Candida glabrata complex
SAMN28991219	G_19_10	Candida glabrata complex
SAMN28991220	G_19_11	Candida glabrata complex
SAMN28991221	G_19_12	Candida glabrata complex
SAMN28991222	G_19_13	Candida glabrata complex
SAMN28991223	G_19_14	Candida glabrata complex
SAMN28991224	G_19_15	Candida glabrata complex
SAMN28991225	G_19_16	Candida glabrata complex
SAMN28991226	G_19_17	Candida glabrata complex
SAMN28991227	G_19_18	Candida glabrata complex
SAMN28991228	G_19_19	Candida glabrata complex
SAMN28991229	G_19_2	Candida glabrata complex
SAMN28991230	G_19_20	Candida glabrata complex
SAMN28991231	G_19_21	Candida glabrata complex
SAMN28991232	G_19_3	Candida glabrata complex
SAMN28991233	G_19_4	Candida glabrata complex
SAMN28991234	G_19_5	Candida glabrata complex
SAMN28991235	G_19_6	Candida glabrata complex

SAMN28991236	G_19_7	Candida glabrata complex
SAMN28991237	G_19_8	Candida glabrata complex
SAMN28991238	G_19_9	Candida glabrata complex
SAMN28991239	G_20_1	Candida glabrata complex
SAMN28991240	G_20_10	Candida glabrata complex
SAMN28991241	G_20_11	Candida glabrata complex
SAMN28991242	G_20_12	Candida glabrata complex
SAMN28991243	G_20_13	Candida glabrata complex
SAMN28991244	G_20_14	Candida glabrata complex
SAMN28991245	G_20_2	Candida glabrata complex
SAMN28991246	G_20_3	Candida glabrata complex
SAMN28991247	G_20_4	Candida glabrata complex
SAMN28991248	G_20_6	Candida glabrata complex
SAMN28991249	G_20_7	Candida glabrata complex
SAMN28991250	G_20_8	Candida glabrata complex
SAMN28991251	G_20_9	Candida glabrata complex
SAMN28991252	G_21_1A	Candida glabrata complex
SAMN28991253	G_21_2	Candida glabrata complex
SAMN28991254	G_21_3	Candida glabrata complex
SAMN28991255	G_21_4	Candida glabrata complex
SAMN28991256	G_21_5A	Candida glabrata complex
SAMN28991257	P_18_1	Candida parapsilosis complex
SAMN28991258	P_18_10	Candida parapsilosis complex
SAMN28991259	P_18_11	Candida parapsilosis complex
SAMN28991260	P_18_2	Candida parapsilosis complex
SAMN28991261	P_18_3	Candida parapsilosis complex
SAMN28991262	P_18_4	Candida parapsilosis complex
SAMN28991263	P_18_5	Candida parapsilosis complex
SAMN28991264	P_18_6	Candida parapsilosis complex
SAMN28991265	P_18_7	Candida parapsilosis complex
SAMN28991266	P_18_8	Candida parapsilosis complex
SAMN28991267	P_18_9	Candida parapsilosis complex
SAMN28991268	P_19_1	Candida parapsilosis complex
SAMN28991269	P_19_11	Candida parapsilosis complex
SAMN28991270	P_19_12	Candida parapsilosis complex
SAMN28991271	P_19_13	Candida parapsilosis complex
SAMN28991272	P_19_14	Candida parapsilosis complex
SAMN28991273	P_19_2	Candida parapsilosis complex
SAMN28991274	P_19_3	Candida parapsilosis complex
SAMN28991275	P_19_4	Candida parapsilosis complex
SAMN28991276	P_19_5	Candida parapsilosis complex
SAMN28991277	P_19_6	Candida parapsilosis complex
SAMN28991278	P_19_7	Candida parapsilosis complex
SAMN28991279	P_19_8	Candida parapsilosis complex

SAMN28991280	P_19_9	Candida parapsilosis complex
SAMN28991281	P_20_1	Candida parapsilosis complex
SAMN28991282	P_20_2	Candida parapsilosis complex
SAMN28991283	P_20_3	Candida parapsilosis complex
SAMN28991284	P_20_4	Candida parapsilosis complex
SAMN28991285	P_20_5	Candida parapsilosis complex
SAMN28991286	P_20_6	Candida parapsilosis complex
SAMN28991287	P_20_7	Candida parapsilosis complex
SAMN28991288	P_20_8	Candida parapsilosis complex
SAMN28991289	P_21_1	Candida parapsilosis complex
SAMN28991290	P_21_2	Candida parapsilosis complex
SAMN28991291	P_21_3	Candida parapsilosis complex
SAMN28991292	T_18_1	Candida tropicalis
SAMN28991293	T_18_10	Candida tropicalis
SAMN28991294	T_18_11	Candida tropicalis
SAMN28991295	T_18_12	Candida tropicalis
SAMN28991296	T_18_13	Candida tropicalis
SAMN28991297	T_18_14	Candida tropicalis
SAMN28991298	T_18_15	Candida tropicalis
SAMN28991299	T_18_16	Candida tropicalis
SAMN28991300	T_18_17	Candida tropicalis
SAMN28991301	T_18_2	Candida tropicalis
SAMN28991302	T_18_3	Candida tropicalis
SAMN28991303	T_18_4	Candida tropicalis
SAMN28991304	T_18_5	Candida tropicalis
SAMN28991305	T_18_6	Candida tropicalis
SAMN28991306	T_18_7	Candida tropicalis
SAMN28991307	T_18_8	Candida tropicalis
SAMN28991308	T_18_9	Candida tropicalis
SAMN28991309	T_19_1	Candida tropicalis
SAMN28991310	T_19_10	Candida tropicalis
SAMN28991311	T_19_11	Candida tropicalis
SAMN28991312	T_19_12	Candida tropicalis
SAMN28991313	T_19_13	Candida tropicalis
SAMN28991314	T_19_14	Candida tropicalis
SAMN28991315	T_19_15	Candida tropicalis
SAMN28991316	T_19_16	Candida tropicalis
SAMN28991317	T_19_17	Candida tropicalis
SAMN28991318	T_19_2	Candida tropicalis
SAMN28991319	T_19_3	Candida tropicalis
SAMN28991320	T_19_4	Candida tropicalis
SAMN28991321	T_19_5	Candida tropicalis
SAMN28991322	T_19_6	Candida tropicalis
SAMN28991323	T_19_7	Candida tropicalis

SAMN28991324	T_19_8	Candida tropicalis
SAMN28991325	T_19_9	Candida tropicalis
SAMN28991326	T_20_1	Candida tropicalis
SAMN28991327	T_20_10	Candida tropicalis
SAMN28991328	T_20_11	Candida tropicalis
SAMN28991329	T_20_12	Candida tropicalis
SAMN28991330	T_20_2	Candida tropicalis
SAMN28991331	T_20_3	Candida tropicalis
SAMN28991332	T_20_4	Candida tropicalis
SAMN28991333	T_20_5	Candida tropicalis
SAMN28991334	T_20_6	Candida tropicalis
SAMN28991335	T_20_7	Candida tropicalis
SAMN28991336	T_20_8	Candida tropicalis
SAMN28991337	T_20_9	Candida tropicalis
SAMN28991338	T_21_1	Candida tropicalis
SAMN28991339	T_21_2	Candida tropicalis
SAMN28991340	T_21_3	Candida tropicalis
SAMN28991341	T_21_4	Candida tropicalis
SAMN28991342	T_21_5	Candida tropicalis
SAMN28991343	T_21_6	Candida tropicalis
SAMN28991344	T_21_7	Candida tropicalis
SAMN28991345	K_19_1	Pichia kudriavzevii
SAMN28991346	K_19_2	Pichia kudriavzevii
SAMN28991347	K_19_3	Pichia kudriavzevii
SAMN28991348	K_20_1	Pichia kudriavzevii
SAMN28991349	K_20_2	Pichia kudriavzevii
SAMN28991350	K_20_3	Pichia kudriavzevii

Supplementary Table 3A: Patient demographics stratified by pathogen

Pathogen	Female		Male		Both genders combined	
	Number of patients	Average age in years (Standard deviation)	Number of patients	Average age in years (Standard deviation)	Number of patients	Average age in years (Standard deviation)
Candida albicans	17	61.0 (19.0)	27	63.3 (19.6)	44	62.4 (19.2)
Candida auris	0	N/A	3	59.3 (19.0)	3	59.3 (19.0)
Candida dubliniensis	2	64.1 (22.6)	3	58.0 (7.2)	5	60.5 (12.9)
Candida glabrata	27	63.7 (18.2)	29	67.3 (12.5)	56	65.6 (15.5)
Candida parapsilosis	11	56.6 (28.1)	25	54.7 (23.3)	36	55.3 (24.5)
Candida tropicalis	21	64.1 (14.1)	32	58.8 (14.9)	53	60.9 (14.7)
Candida krusei	1	29.6 (N/A)	5	49.0 (28.6)	6	45.8 (26.7)
**All organisms**	**79**	**61.8 (19.0)**	**124**	**60.5 (18.4)**	**203**	**61.0 (18.6)**

Supplementary Table 3B: Patient demographics of *C. parapsilosis* and *C. tropicalis* stratified by clustering of isolates

Clustering of isolates	Female		Male		Both genders combined	
	Number of patients	Average age in years (Standard deviation)	Number of patients	Average age in years (Standard deviation)	Number of patients	Average age in years (Standard deviation)
Candida parapsilosis						
All *C. parapsilosis* isolates	11	56.6 (28.1)	25	54.7 (23.3)	36	55.3 (24.5)
Clustered isolates	6	64.1 (19.0)	13	60.7 (17.8)	19	61.8 (27.7)
C. parapsilosis Cluster 1	4	71.5 (3.7)	2	57.6 (26.4)	6	66.9 (14.1)
C. parapsilosis Cluster 2	0	N/A	7	62.2 (17.4)	7	62.2 (17.4)
C. parapsilosis Cluster 3	1	25.8 (N/A)	3	68.8 (10.8)	4	58.0 (23.3)
C. parapsilosis Cluster 4	1	72.9 (N/A)	1	31.9 (N/A)	2	52.4 (29.)
Unclustered isolates	5	47.6 (36.5)	12	48.3 (27.4)	17	48.1 (29.2)
Candida tropicalis						
All *C. tropicalis* isolates	21	64.1 (14.1)	32	58.8 (14.9)	53	60.9 (14.7)
Clustered	5	48.0 (13.4)	1	73.2 (N/A)	6	52.2 (15.8)
Unclustered	16	69.2 (10.2)	31	58.4 (14.9)	47	62.1 (14.3)
**Grand Total**	**32**	**61.6 (19.9)**	**57**	**57.0 (19.0)**	**89**	**58.7 (19.3)**

N/A: Not applicable

Supplementary Table 4: Cause of candidaemia distributed by primary treating teams

Primary treating team	*C. albicans*		*C. auris*		*C. dubliniensis*		*C. glabrata*		*C. parapsilosis*		*C.tropicalis*		*C. krusei*		All isolates	
Bone Marrow Transplant							1	1.8%	2	5.6%	1	1.9%	3	50.0%	7	3.4%
Cardiac Surgery	3	6.8%					2	3.6%	2	5.6%	3	5.7%	1	16.7%	11	5.4%
Cardiology	3	6.8%			1	20.0%	2	3.6%	2	5.6%	1	1.9%			9	4.4%
Cardiothoracic/vascular surgery (Paediatrics)									1	2.8%					1	0.5%
Colorectal Surgery	5	11.4%					3	5.4%	1	2.8%					9	4.4%
Emergency Medicine	1	2.3%					2	3.6%	2	5.6%	1	1.9%			6	3.0%
Endocrinology	1	2.3%					1	1.8%	1	2.8%					3	1.5%
ENT Surgery			1	33.3%											1	0.5%
Gastroenterology	3	6.8%			1	20.0%	1	1.8%			1	1.9%			6	3.0%
General Medicine	1	2.3%					2	3.6%	1	2.8%	2	3.8%			6	3.0%
General Surgery	1	2.3%					1	1.8%			1	1.9%			3	1.5%
Haematology	1	2.3%					10	17.9%	4	11.1%	18	34.0%			33	16.3%
Hepatobiliary Surgery									1	2.8%					1	0.5%
Hepatobiliary Surgery (Cancer)	1	2.3%					3	5.4%	1	2.8%					5	2.5%
Infectious Diseases	1	2.3%	1	33.3%			3	5.4%			4	7.5%	1	16.7%	10	4.9%
Liver Transplant Team	2	4.5%	1	33.3%											3	1.5%
Medical Oncology	4	9.1%			1	20.0%	7	12.5%	2	5.6%	6	11.3%			20	9.9%
Medical Oncology (Paediatric)	2	4.5%					1	1.8%	3	8.3%			1	16.7%	7	3.4%
Neonatology									1	2.8%					1	0.5%
Nephrology	2	4.5%			1	20.0%	3	5.4%	5	13.9%	4	7.5%			15	7.4%
Neurology											1	1.9%			1	0.5%
Neurology (Stroke)	1	2.3%									2	3.8%			3	1.5%
Neurosurgery									4	11.1%					4	2.0%
Obstetrics & Gynaecology							1	1.8%							1	0.5%

Paediatric Medicine	1	2.3%													1	0.5%
Renal Transplant Team	1	2.3%					2	3.6%	1	2.8%					4	2.0%
Respiratory Medicine	5	11.4%			1	20.0%	3	5.4%	1	2.8%	3	5.7%			13	6.4%
Rheumatology							1	1.8%							1	0.5%
Sub-Acute Medicine							1	1.8%							1	0.5%
Thoracic Surgery									1	2.8%					1	0.5%
Trauma							1	1.8%			3	5.7%			4	2.0%
Upper GI Surgery							2	3.6%							2	1.0%
Urology	5	11.4%					2	3.6%			1	1.9%			8	3.9%
Vascular surgery							1	1.8%			1	1.9%			2	1.0%

Supplementary Table 5: Primary treating team for clustered and unclustered *C. parapsilosis* and *C. tropicalis* isolates

Primary treating team	*Candida parapsilosis*														*Candida tropicalis*					
	Clustered										Unclustered		All patients		Clustered		Unclustered		All patients	
	Parapsilosis Cluster 1		Parapsilosis Cluster 2		Parapsilosis Cluster 3		Parapsilosis Cluster 4		All clustered patients											
Bone Marrow Transplant			1	14.29%					1	5.26%	1	5.88%	2	5.56%			1	2.13%	1	1.89%
Cardiac Surgery					1	25.00%			1	5.26%	1	5.88%	2	5.56%			3	6.38%	3	5.66%
Cardiology					2	50.00%			2	10.53%			2	5.56%			1	2.13%	1	1.89%
Cardiothoracic/vascular surgery (Paediatrics)											1	5.88%	1	2.78%						
Colorectal Surgery	1	16.67%							1	5.26%			1	2.78%						
Emergency Medicine							1	50.00%	1	5.26%	1	5.88%	2	5.56%			1	2.13%	1	1.89%
Endocrinology	1	16.67%							1	5.26%			1	2.78%						
ENT Surgery																				
Gastroenterology																	1	2.13%	1	1.89%
General Medicine			1	14.29%					1	5.26%			1	2.78%			2	4.26%	2	3.77%
General Surgery																	1	2.13%	1	1.89%
Haematology											4	23.53%	4	11.11%	5	83.33%	13	27.66%	18	33.96%
Hepatobiliary Surgery											1	5.88%	1	2.78%						
Hepatobiliary Surgery (Cancer)	1	16.67%							1	5.26%			1	2.78%						
Infectious Diseases																	4	8.51%	4	7.55%
Liver Transplant Team																				
Medical Oncology	1	16.67%							1	5.26%	1	5.88%	2	5.56%			6	12.77%	6	11.32%
Medical Oncology (Paediatric)											3	17.65%	3	8.33%						
Neonatology											1	5.88%	1	2.78%						
Nephrology	1	16.67%	3	42.86%					4	21.05%	1	5.88%	5	13.89%			4	8.51%	4	7.55%
Neurology															1	16.67%			1	1.89%
Neurology (Stroke)																	2	4.26%	2	3.77%

Neurosurgery	1	16.67%	2	28.57%			1	50.00%	4	21.05%			4	11.11%						
Obstetrics & Gynaecology																				
Paediatric Medicine																				
Renal Transplant Team											1	5.88%	1	2.78%						
Respiratory Medicine											1	5.88%	1	2.78%			3	6.38%	3	5.66%
Rheumatology																				
Sub-Acute Medicine																				
Thoracic Surgery					1	25.00%			1	5.26%			1	2.78%						
Trauma																	3	6.38%	3	5.66%
Upper GI Surgery																				
Urology																	1	2.13%	1	1.89%
Vascular surgery																	1	2.13%	1	1.89%

Supplementary Table 6: Primary treating team for *C. glabrata* isolates stratified by MLST

**Primary treating team**	**Sequence types with >5 isolates**								**Combined (ST 7, 55, 59, 83)**		**All others (Sequence Types with <5 isolates)**		**All isolates**	
	**ST 55**		**ST 59**		**ST 7**		**ST 83**							
Bone Marrow Transplant			1	14.3%					1	2.6%			1	1.8%
Cardiac Surgery	1	10.0%					1	14.3%	2	5.1%			2	3.6%
Cardiology					1	6.7%			1	2.6%	1	5.9%	2	3.6%
Colorectal Surgery			1	14.3%					2	5.1%	1	5.9%	3	5.4%
Emergency Medicine	1	10.0%			1	6.7%			2	5.1%			2	3.6%
Endocrinology			1	14.3%					1	2.6%			1	1.8%
Gastroenterology					1	6.7%			1	2.6%			1	1.8%
General Medicine					1	6.7%			1	2.6%	1	5.9%	2	3.6%
General Surgery					1	6.7%			1	2.6%			1	1.8%
Haematology	1	10.0%	3	42.9%	2	13.3%			6	15.4%	4	23.5%	10	17.9%
Hepatobiliary Surgery (Cancer)	1	10.0%					1	14.3%	2	5.1%	1	5.9%	3	5.4%
Infectious Diseases	1	10.0%			1	6.7%			1	2.6%	2	11.8%	3	5.4%
Medical Oncology	2	20.0%			2	13.3%	1	14.3%	5	12.8%	2	11.8%	7	12.5%
Medical Oncology (Paediatric)										0.0%	1	5.9%	1	1.8%
Nephrology			1	14.3%	2	13.3%			3	7.7%			3	5.4%
Obstetrics & Gynaecology	1	10.0%							1	2.6%			1	1.8%
Renal Transplant Team					2	13.3%			2	5.1%			2	3.6%
Respiratory Medicine					1	6.7%	1	14.3%	2	5.1%	1	5.9%	3	5.4%
Rheumatology	1	10.0%							1	2.6%			1	1.8%
Sub-Acute Medicine							1	14.3%	1	2.6%			1	1.8%
Trauma	1	10.0%							1	2.6%			1	1.8%
Upper GI Surgery							1	14.3%	1	2.6%	1	5.9%	2	3.6%
Urology										0.0%	2	11.8%	2	3.6%
Vascular surgery							1	14.3%	1	2.6%			1	1.8%
Grand Total	10	100.0%	8	100.0%	15	100.0%	7	100.0%	39	100.0%	17	100.0%	56	100.0%

Supplementary Table 7. *C. glabrata* PDR1 mutations in azole resistance and sensitive isolates in comparison to *C. glabrata* CBS138

			Amino acid position in PDR1																
S/ N	Isolate name	MIC (mg/L)	76	91	98	139	143	189	243	253	259	282	291	368	732	745	555	821	935
1	G_18_3	0.25	Ser76 Pro	Val91 Ile	Leu98 Ser		Thr143 Pro												
2	G_21_2	0.38	Ser76 Pro	Val91 Ile	Leu98 Ser	Leu13 9Ile	Thr143 Pro												
3	G_21_5	0.5	Ser76 Pro	Val91 Ile	Leu98 Ser		Thr143 Pro												
4	G_21_4	1	Ser76 Pro	Val91 Ile	Leu98 Ser	Leu13 9Ile	Thr143 Pro												
5	G_20_11	1.5	Ser76 Pro	Val91 Ile	Leu98 Ser		Thr143 Pro												
6	G_18_2	2	Ser76 Pro	Val91 Ile	Leu98 Ser		Thr143 Pro	Gly189 Val											
7	G_18_4	2	Ser76 Pro	Val91 Ile	Leu98 Ser		Thr143 Pro				Glu259 Gly								
8	G_18_5	2	Ser76 Pro	Val91 Ile	Leu98 Ser	Leu13 9Ile	Thr143 Pro												
9	G_19_10	2	Ser76 Pro	Val91 Ile	Leu98 Ser		Thr143 Pro												
10	G_19_18	2	Ser76 Pro	Val91 Ile	Leu98 Ser		Thr143 Pro												
11	G_19_20	2	Ser76 Pro	Val91 Ile	Leu98 Ser		Thr143 Pro												
12	G_19_21	2	Ser76 Pro	Val91 Ile	Leu98 Ser		Thr143 Pro												
13	G_21_3	2	Ser76 Pro	Val91 Ile	Leu98 Ser		Thr143 Pro												
14	G_18_7	3	Ser76 Pro	Val91 Ile	Leu98 Ser		Thr143 Pro												
15	G_18_8	3	Ser76 Pro	Val91 Ile	Leu98 Ser	Leu13 9Ile	Thr143 Pro												
16	G_19_17	3	Ser76 Pro	Val91 Ile	Leu98 Ser	Leu13 9Ile	Thr143 Pro												
17	G_20_2	3	Ser76 Pro	Val91 Ile	Leu98 Ser	Leu13 9Ile	Thr143 Pro									Thr745 Ala			
18	G_20_3	3	Ser76 Pro	Val91 Ile	Leu98 Ser	Leu13 9Ile	Thr143 Pro												
19	G_20_4	3	Ser76 Pro	Val91 Ile	Leu98 Ser		Thr143 Pro												
20	G_19_19	4	Ser76 Pro	Val91 Ile	Leu98 Ser	Leu13 9Ile	Thr143 Pro											Ala821 Thr	

21	G_20_1	4	Ser76<br>Pro	Val91<br>Ile	Leu98<br>Ser	Leu13<br>9Ile	Thr143<br>Pro				Glu259<br>Gly								
22	G_21_1<br>A	4	Ser76<br>Pro	Val91<br>Ile	Leu98<br>Ser		Thr143<br>Pro												
23	G_19_12	6	Ser76<br>Pro	Val91<br>Ile	Leu98<br>Ser		Thr143<br>Pro									Thr745<br>Ala			
24	G_19_5	6	Ser76<br>Pro	Val91<br>Ile	Leu98<br>Ser		Thr143<br>Pro									Thr745<br>Ala			
25	G_18_16	6	Ser76<br>Pro	Val91<br>Ile	Leu98<br>Ser		Thr143<br>Pro			Leu253<br>Ser	Glu259<br>Gly								
26	G_19_13	6	Ser76<br>Pro	Val91<br>Ile	Leu98<br>Ser		Thr143<br>Pro												
27	G_19_14	6	Ser76<br>Pro	Val91<br>Ile	Leu98<br>Ser		Thr143<br>Pro												
28	G_19_15	6	Ser76<br>Pro	Val91<br>Ile	Leu98<br>Ser		Thr143<br>Pro				Glu259<br>Gly								
29	G_19_7	6	Ser76<br>Pro	Val91<br>Ile	Leu98<br>Ser		Thr143<br>Pro				Glu259<br>Gly								
30	G_20_10	6	Ser76<br>Pro	Val91<br>Ile	Leu98<br>Ser		Thr143<br>Pro												
31	G_20_12	6	Ser76<br>Pro	Val91<br>Ile	Leu98<br>Ser	Leu13<br>9Ile	Thr143<br>Pro												
32	G_20_9	6	Ser76<br>Pro	Val91<br>Ile	Leu98<br>Ser		Thr143<br>Pro												
33	G_19_4	6	Ser76<br>Pro	Val91<br>Ile	Leu98<br>Ser		Thr143<br>Pro												
34	G_19_11	8	Ser76<br>Pro	Val91<br>Ile	Leu98<br>Ser		Thr143<br>Pro										Glu555<br>Asp		
35	G_18_14	8	Ser76<br>Pro	Val91<br>Ile	Leu98<br>Ser		Thr143<br>Pro				Glu259<br>Gly								
36	G_18_15	8	Ser76<br>Pro	Val91<br>Ile	Leu98<br>Ser		Thr143<br>Pro												
37	G_19_2	12		Val91<br>Ile	Leu98<br>Ser		Thr143<br>Pro		Asp243<br>Asn					Lys368<br>Gln					
38	G_18_13	12	Ser76<br>Pro	Val91<br>Ile	Leu98<br>Ser		Thr143<br>Pro									Thr745<br>Ala			
39	G_18_1	12	Ser76<br>Pro	Val91<br>Ile	Leu98<br>Ser		Thr143<br>Pro				Glu259<br>Gly								
40	G_18_10	12	Ser76<br>Pro	Val91<br>Ile	Leu98<br>Ser		Thr143<br>Pro												
41	G_19_3	12	Ser76<br>Pro	Val91<br>Ile	Leu98<br>Ser		Thr143<br>Pro												
42	G_19_9	12	Ser76<br>Pro	Val91<br>Ile	Leu98<br>Ser		Thr143<br>Pro												
43	G_20_7	12	Ser76<br>Pro	Val91<br>Ile	Leu98<br>Ser	Leu13<br>9Ile	Thr143<br>Pro												
44	G_19_1	16	Ser76<br>Pro	Val91<br>Ile	Leu98<br>Ser		Thr143<br>Pro												

45	G_19_6	16	Ser76 Pro	Val91 Ile	Leu98 Ser		Thr143 Pro				Glu259 Gly								Leu935 Met
46	G_19_16	24	Ser76 Pro	Val91 Ile	Leu98 Ser		Thr143 Pro												
47	G_18_11	32	Ser76 Pro	Val91 Ile	Leu98 Ser		Thr143 Pro												
48	G_18_12	32	Ser76 Pro	Val91 Ile	Leu98 Ser		Thr143 Pro												
49	G_18_9	32	Ser76 Pro	Val91 Ile	Leu98 Ser		Thr143 Pro												
50	G_20_8	>256	Ser76 Pro	Val91 Ile			Thr143 Pro								Leu732 Ser				
51	G_18_6	>256	Ser76 Pro	Val91 Ile	Leu98 Ser		Thr143 Pro									Thr745 Ala			
52	G_19_8	>256	Ser76 Pro		Leu98 Ser		Thr143 Pro						Leu291 Gln			Thr745 Ala			
53	G_20_6	>256	Ser76 Pro	Val91 Ile	Leu98 Ser		Thr143 Pro					Arg282 Gly				Thr745 Ala			
54	G_20_14	>256		Val91 Ile	Leu98 Ser		Thr143 Pro												

Supplementary Table 8: MLST and allelic profiles
*** represents the nearest sequence type**

C. albicans	ST	Allelic profile						
A_18_1	2403*	AAT1a(53)	ACC1(9)	ADP1(43)	MPIb(14)	SYA1(24)	VPS13(20)	ZWF1b(107)
A_18_10	3205*	AAT1a(53)	ACC1(9)	ADP1(10)	MPIb(36)	SYA1(~159)	VPS13(182)	ZWF1b(~10)
A_18_11	358*	AAT1a(8)	ACC1(3)	ADP1(2)	MPIb(9)	SYA1(2)	VPS13(20)	ZWF1b(~62)
A_18_12	1476*	AAT1a(77)	ACC1(3)	ADP1(6)	MPIb(14)	SYA1(34)	VPS13(45)	ZWF1b(161)
A_18_13	721*	AAT1a(8)	ACC1(2)	ADP1(2)	MPIb(4)	SYA1(2)	VPS13(24)	ZWF1b(~62)
A_18_14	617*	AAT1a(13)	ACC1(8)	ADP1(6)	MPIb(19)	SYA1(30)	VPS13(32)	ZWF1b(22)
A_18_15	2016*	AAT1a(4)	ACC1(7)	ADP1(6)	MPIb(9)	SYA1(2)	VPS13(~205)	ZWF1b(-)
A_18_2	1527*	AAT1a(3)	ACC1(3)	ADP1(6)	MPIb(4)	SYA1(2)	VPS13(20)	ZWF1b(27)
A_18_3	1446*	AAT1a(13)	ACC1(8)	ADP1(6)	MPIb(9)	SYA1(2)	VPS13(4)	ZWF1b(12)
A_18_4	617*	AAT1a(153?)	ACC1(8)	ADP1(6)	MPIb(19)	SYA1(30)	VPS13(32)	ZWF1b(22)
A_18_5	2745*	AAT1a(~77)	ACC1(7)	ADP1(14)	MPIb(14)	SYA1(34)	VPS13(45)	ZWF1b(271)
A_18_6	3633*	AAT1a(8)	ACC1(40)	ADP1(6)	MPIb(~97)	SYA1(53)	VPS13(20)	ZWF1b(~271)
A_18_7	3622*	AAT1a(4)	ACC1(8)	ADP1(6)	MPIb(4)	SYA1(34)	VPS13(24)	ZWF1b(35)
A_18_8	629*	AAT1a(13)	ACC1(3)	ADP1(6)	MPIb(4)	SYA1(53)	VPS13(45)	ZWF1b(284)
A_19_1	105*	AAT1a(33)	ACC1(13)	ADP1(6)	MPIb(~97)	SYA1(18)	VPS13(47)	ZWF1b(12)
A_19_10	215*	AAT1a(33)	ACC1(13)	ADP1(14)	MPIb(~97)	SYA1(~34)	VPS13(20)	ZWF1b(12)
A_19_11	3227*	AAT1a(24)	ACC1(7)	ADP1(10)	MPIb(9)	SYA1(29)	VPS13(20)	ZWF1b(~55)
A_19_12	2100*	AAT1a(14)	ACC1(7)	ADP1(6)	MPIb(4)	SYA1(76)	VPS13(3)	ZWF1b(35)
A_19_13	3109*	AAT1a(53)	ACC1(3)	ADP1(6)	MPIb(4)	SYA1(97)	VPS13(3)	ZWF1b(12)
A_19_14	89*	AAT1a(14)	ACC1(19)	ADP1(14)	MPIb(4)	SYA1(76)	VPS13(20)	ZWF1b(~297)
A_19_15	660*	AAT1a(24)	ACC1(7)	ADP1(14)	MPIb(19)	SYA1(34)	VPS13(55)	ZWF1b(10)
A_19_16	2061*	AAT1a(13)	ACC1(13)	ADP1(6)	MPIb(5)	SYA1(34)	VPS13(126)	ZWF1b(271)
A_19_17	061*	AAT1a(14)	ACC1(13)	ADP1(6)	MPIb(~97)	SYA1(34)	VPS13(47)	ZWF1b(~297)
A_19_18	527*	AAT1a(24)	ACC1(3)	ADP1(6)	MPIb(4)	SYA1(2)	VPS13(20)	ZWF1b(22)
A_19_2	2340*	AAT1a(4)	ACC1(3)	ADP1(10)	MPIb(4)	SYA1(2)	VPS13(~205)	ZWF1b(161)
A_19_4	2054*	AAT1a(33)	ACC1(7)	ADP1(14)	MPIb(14)	SYA1(34)	VPS13(45)	ZWF1b(22)

A_19_5	2051*	AAT1a(24)	ACC1(7)	ADP1(6)	MPIb(9)	SYA1(24)	VPS13(20)	ZWF1b(136)
A_19_6	1460*	AAT1a(~152)	ACC1(2)	ADP1(6)	MPIb(4)	SYA1(67)	VPS13(279)	ZWF1b(12)
A_19_7	2054*	AAT1a(33)	ACC1(3)	ADP1(6)	MPIb(~14)	SYA1(34)	VPS13(45)	ZWF1b(271)
A_19_8	1089*	AAT1a(24)	ACC1(3)	ADP1(6)	MPIb(9)	SYA1(2)	VPS13(20)	ZWF1b(161)
A_19_9	1912*	AAT1a(~159)	ACC1(7)	ADP1(6)	MPIb(4)	SYA1(25)	VPS13(32)	ZWF1b(22)
A_20_1	3548*	AAT1a(33)	ACC1(7)	ADP1(10)	MPIb(5)	SYA1(2)	VPS13(126)	ZWF1b(271)
A_20_2	759*	AAT1a(53)	ACC1(3)	ADP1(10)	MPIb(9)	SYA1(67)	VPS13(205)	ZWF1b(-)
A_20_3	1525*	AAT1a(77)	ACC1(3)	ADP1(14)	MPIb(14)	SYA1(34)	VPS13(45)	ZWF1b(22)
A_20_4	137*	AAT1a(4)	ACC1(2)	ADP1(6)	MPIb(9)	SYA1(29)	VPS13(205)	ZWF1b(12)
A_20_5	1476*	AAT1a(33)	ACC1(3)	ADP1(6)	MPIb(14)	SYA1(24)	VPS13(45)	ZWF1b(161)
A_20_6	380*	AAT1a(8)	ACC1(3)	ADP1(6)	MPIb(~9)	SYA1(2)	VPS13(24)	ZWF1b(12)
A_20_7	101*	AAT1a(13)	ACC1(3)	ADP1(106)	MPIb(4)	SYA1(~52)	VPS13(32)	ZWF1b(12)
A_21_1	2192*	AAT1a(33)	ACC1(8)	ADP1(14)	MPIb(~97)	SYA1(25)	VPS13(20)	ZWF1b(~297)
A_21_2	2629*	AAT1a(4)	ACC1(2)	ADP1(6)	MPIb(9)	SYA1(~14)	VPS13(32)	ZWF1b(22)
A_21_4	1671*	AAT1a(4)	ACC1(19)	ADP1(6)	MPIb(~97)	SYA1(18)	VPS13(20)	ZWF1b(~297)
C. krusei	**ST**	**Allelic profile**						
K_19_1	37*	HIS3(4)	LEU2(10)	NMT1(13)	TRP1(12)	ADE2(4)	LYS2D(2)	
K_19_2	59*	HIS3(4)	LEU2(10)	NMT1(~33)	TRP1(12)	ADE2(16)	LYS2D(2)	
K_19_3	66*	HIS3(11)	LEU2(1)	NMT1(1)	TRP1(9)	ADE2(4)	LYS2D(2)	
K_20_1	59*	HIS3(4)	LEU2(1)	NMT1(1)	TRP1(~9)	ADE2(16)	LYS2D(2)	
K_20_2	211*	HIS3(2)	LEU2(~1)	NMT1(1)	TRP1(22)	ADE2(4)	LYS2D(~1)	
K_20_3	66*	HIS3(11)	LEU2(1)	NMT1(1)	TRP1(9)	ADE2(4)	LYS2D(2)	
C. tropicalis	**ST**	**Allelic profile**						
T_18_1	-	ICL1(26)	MDR1(117)	SAPT2(22)	SAPT4(7)	XYR1(~201)	ZWF1a(1)	
T_18_10	-	ICL1(37)	MDR1(7)	SAPT2(3)	SAPT4(~91)	XYR1(~202)	ZWF1a(1)	
T_18_11	612	ICL1(1)	MDR1(7)	SAPT2(4)	SAPT4(7)	XYR1(85)	ZWF1a(7)	
T_18_12	-	ICL1(1)	MDR1(33)	SAPT2(22)	SAPT4(14?)	XYR1(~202)	ZWF1a(66)	

T_18_13	-	ICL1(1)	MDR1(7)	SAPT2(3)	SAPT4(7)	XYR1(~201)	ZWF1a(1)
T_18_14	-	ICL1(1)	MDR1(~187)	SAPT2(3)	SAPT4(7)	XYR1(~201)	ZWF1a(1)
T_18_15	-	ICL1(1)	MDR1(117)	SAPT2(4)	SAPT4(7)	XYR1(~202)	ZWF1a(1)
T_18_16	-	ICL1(10)	MDR1(7)	SAPT2(3)	SAPT4(3)	XYR1(~201)	ZWF1a(7)
T_18_17	-	ICL1(1)	MDR1(122)	SAPT2(3)	SAPT4(7)	XYR1(~85)	ZWF1a(1)
T_18_2	-	ICL1(1)	MDR1(9)	SAPT2(3)	SAPT4(8)	XYR1(~141)	ZWF1a(1)
T_18_3	-	ICL1(1)	MDR1(117)	SAPT2(4)	SAPT4(7)	XYR1(~202)	ZWF1a(
T_18_4	-	ICL1(31)	MDR1(7)	SAPT2(4)	SAPT4(8)	XYR1(~208)	ZWF1a(48)
T_18_5	-	ICL1(10)	MDR1(7)	SAPT2(4)	SAPT4(91)	XYR1(~179)	ZWF1a(10)
T_18_6	-	ICL1(1)	MDR1(152)	SAPT2(4)	SAPT4(~126)	XYR1(~201)	ZWF1a(7)
T_18_7	-	ICL1(1)	MDR1(~111)	SAPT2(4)	SAPT4(8)	XYR1(92)	ZWF1a(7)
T_18_8	-	ICL1(26)	MDR1(~13)	SAPT2(4)	SAPT4(5)	XYR1(92)	ZWF1a(7)
T_18_9	-	ICL1(1)	MDR1(13)	SAPT2(22)	SAPT4(~14)	XYR1(~208)	ZWF1a(2)
T_19_1	-	ICL1(~10)	MDR1(7)	SAPT2(3)	SAPT4(7)	XYR1(~185)	ZWF1a(1)
T_19_10	-	ICL1(1)	MDR1(9)	SAPT2(22)	SAPT4(8)	XYR1(85)	ZWF1a(2)
T_19_11	535	ICL1(1)	MDR1(7)	SAPT2(3)	SAPT4(3)	XYR1(92)	ZWF1a(1)
T_19_12	-	ICL1(20?)	MDR1(69)	SAPT2(3)	SAPT4(3)	XYR1(~202)	ZWF1a(1)
T_19_13	-	ICL1(1)	MDR1(33)	SAPT2(4)	SAPT4(126)	XYR1(~202)	ZWF1a(66)
T_19_14	-	ICL1(31)	MDR1(7)	SAPT2(3)	SAPT4(3)	XYR1(~203)	ZWF1a(2)
T_19_15	-	ICL1(1)	MDR1(7)	SAPT2(3)	SAPT4(7)	XYR1(~201)	ZWF1a(7)
T_19_16	-	ICL1(1)	MDR1(~9)	SAPT2(3)	SAPT4(~126)	XYR1(85)	ZWF1a(1)
T_19_2	-	ICL1(26)	MDR1(7)	SAPT2(3)	SAPT4(52)	XYR1(~201)	ZWF1a(2)
T_19_3	-	ICL1(1)	MDR1(32)	SAPT2(3)	SAPT4(~126)	XYR1(~201)	ZWF1a(1)
T_19_4	-	ICL1(1)	MDR1(7)	SAPT2(3)	SAPT4(33)	XYR1(~201)	ZWF1a(7)
T_19_5	-	ICL1(1)	MDR1(100)	SAPT2(3)	SAPT4(3)	XYR1(~185)	ZWF1a(1)
T_19_6	-	ICL1(10)	MDR1(117)	SAPT2(~59)	SAPT4(7)	XYR1(~16)	ZWF1a(1)
T_19_7	-	ICL1(1)	MDR1(9)	SAPT2(22)	SAPT4(~126)	XYR1(85)	ZWF1a(2)
T_19_8	-	ICL1(1)	MDR1(152)	SAPT2(3)	SAPT4(5)	XYR1(~201)	ZWF1a(10)
T_19_9	-	ICL1(1)	MDR1(32)	SAPT2(3)	SAPT4(3)	XYR1(~195)	ZWF1a(1)

T_20_1	-	ICL1(26)	MDR1(7)	SAPT2(3)	SAPT4(3)	XYR1(~201)	ZWF1a(2)
T_20_10	-	ICL1(1)	MDR1(~19)	SAPT2(4)	SAPT4(14)	XYR1(85)	ZWF1a(1)
T_20_11	-	ICL1(26)	MDR1(7)	SAPT2(22)	SAPT4(3)	XYR1(~201)	ZWF1a(48)
T_20_12	-	ICL1(1)	MDR1(152)	SAPT2(22)	SAPT4(126?)	XYR1(85)	ZWF1a(1)
T_20_2	-	ICL1(1)	MDR1(9)	SAPT2(~3)	SAPT4(8)	XYR1(~203)	ZWF1a(1)
T_20_3	-	ICL1(1)	MDR1(9)	SAPT2(~64)	SAPT4(8)	XYR1(~141)	ZWF1a(1)
T_20_4	-	ICL1(1)	MDR1(7)	SAPT2(22)	SAPT4(52)	XYR1(~201)	ZWF1a(48)
T_20_5	-	ICL1(10)	MDR1(~142)	SAPT2(22)	SAPT4(~132)	XYR1(85)	ZWF1a(1)
T_20_6	-	ICL1(1)	MDR1(9)	SAPT2(4)	SAPT4(36)	XYR1(~201)	ZWF1a(18)
T_20_7	-	ICL1(26)	MDR1(7)	SAPT2(22)	SAPT4(~5)	XYR1(~201)	ZWF1a(1)
T_20_8	-	ICL1(1)	MDR1(7)	SAPT2(3)	SAPT4(~126)	XYR1(~194)	ZWF1a(7)
T_20_9	-	ICL1(1)	MDR1(19)	SAPT2(22)	SAPT4(14)	XYR1(85)	ZWF1a(7)
T_21_1	-	ICL1(26)	MDR1(7)	SAPT2(3)	SAPT4(3)	XYR1(~201)	ZWF1a(48)
T_21_2	-	ICL1(~31)	MDR1(33)	SAPT2(4)	SAPT4(7)	XYR1(~201)	ZWF1a(2)
T_21_3	-	ICL1(1)	MDR1(~195)	SAPT2(4)	SAPT4(3)	XYR1(~203)	ZWF1a(1)
T_21_4	-	ICL1(1)	MDR1(9)	SAPT2(3)	SAPT4(~132)	XYR1(~201)	ZWF1a(7)
T_21_5	-	ICL1(1)	MDR1(7)	SAPT2(4)	SAPT4(91)	XYR1(85)	ZWF1a(7)
T_21_6	-	ICL1(26)	MDR1(7)	SAPT2(22)	SAPT4(52)	XYR1(~201)	ZWF1a(7)
T_21_7	-	ICL1(26)	MDR1(7)	SAPT2(3)	SAPT4(3)	XYR1(~201)	ZWF1a(2)
C. glabrata	**ST**	**Allelic profile**					
G_20_4	7	FKS(3)	LEU2(4)	NMT1(4)	TRP1(3)	UGP1(3)	URA3(4)
G_20_14	7	FKS(3)	LEU2(4)	NMT1(4)	TRP1(3)	UGP1(3)	URA3(4)
G_20_10	7	FKS(3)	LEU2(4)	NMT1(4)	TRP1(3)	UGP1(3)	URA3(4)
G_19_3	7	FKS(3)	LEU2(4)	NMT1(4)	TRP1(3)	UGP1(3)	URA3(4)
G_19_21	7	FKS(3)	LEU2(4)	NMT1(4)	TRP1(3)	UGP1(3)	URA3(4)
G_18_11	7	FKS(3)	LEU2(4)	NMT1(4)	TRP1(3)	UGP1(3)	URA3(4)
G_19_16	7	FKS(3)	LEU2(4)	NMT1(4)	TRP1(3)	UGP1(3)	URA3(4)
G_19_14	7	FKS(3)	LEU2(4)	NMT1(4)	TRP1(3)	UGP1(3)	URA3(4)

G_19_9	7	FKS(3)	LEU2(4)	NMT1(4)	TRP1(3)	UGP1(3)	URA3(4)
G_21_3	7	FKS(3)	LEU2(4)	NMT1(4)	TRP1(3)	UGP1(3)	URA3(4)
G_19_20	7	FKS(3)	LEU2(4)	NMT1(4)	TRP1(3)	UGP1(3)	URA3(4)
G_18_15	7	FKS(3)	LEU2(4)	NMT1(4)	TRP1(3)	UGP1(3)	URA3(4)
G_20_9	7	FKS(3)	LEU2(4)	NMT1(4)	TRP1(3)	UGP1(3)	URA3(4)
G_19_18	7	FKS(3)	LEU2(4)	NMT1(4)	TRP1(3)	UGP1(3)	URA3(4)
G_18_9	7	FKS(3)	LEU2(4)	NMT1(4)	TRP1(3)	UGP1(3)	URA3(4)
G_21_5B	24*, 21*	FKS(6)	LEU2(16)	NMT1(~10)	TRP1(4)	UGP1(3)	URA3(11)
G_21_5	24*, 21*	FKS(6)	LEU2(16)	NMT1(~10)	TRP1(4)	UGP1(3)	URA3(11)
G_18_12	55	FKS(3)	LEU2(6)	NMT1(22)	TRP1(2)	UGP1(3)	URA3(9)
G_18_7	55	FKS(3)	LEU2(6)	NMT1(22)	TRP1(2)	UGP1(3)	URA3(9)
G_19_6	55	FKS(3)	LEU2(6)	NMT1(22)	TRP1(2)	UGP1(3)	URA3(9)
G_18_1	55	FKS(3)	LEU2(6)	NMT1(22)	TRP1(2)	UGP1(3)	URA3(9)
G_18_14	55	FKS(3)	LEU2(6)	NMT1(22)	TRP1(2)	UGP1(3)	URA3(9)
G_19_7	55	FKS(3)	LEU2(6)	NMT1(22)	TRP1(2)	UGP1(3)	URA3(9)
G_19_15	55	FKS(3)	LEU2(6)	NMT1(22)	TRP1(2)	UGP1(3)	URA3(9)
G_20_1	55	FKS(3)	LEU2(6)	NMT1(22)	TRP1(2)	UGP1(3)	URA3(9)
G_18_16	55	FKS(3)	LEU2(6)	NMT1(22)	TRP1(2)	UGP1(3)	URA3(9)
G_18_4	55	FKS(3)	LEU2(6)	NMT1(22)	TRP1(2)	UGP1(3)	URA3(9)
G_20_8	78	FKS(26)	LEU2(4)	NMT1(38)	TRP1(28)	UGP1(14)	URA3(23)
G_19_4	19*	FKS(~6)	LEU2(6)	NMT1(5)	TRP1(2)	UGP1(3)	URA3(4)
G_19_10	95	FKS(6)	LEU2(10)	NMT1(5)	TRP1(2)	UGP1(3)	URA3(4)
G_20_11	46	FKS(20)	LEU2(13)	NMT1(22)	TRP1(9)	UGP1(3)	URA3(2)
G_18_8	46	FKS(20)	LEU2(13)	NMT1(22)	TRP1(9)	UGP1(3)	URA3(2)
G_18_13	59	FKS(7)	LEU2(13)	NMT1(17)	TRP1(9)	UGP1(3)	URA3(19)
G_19_5	59	FKS(7)	LEU2(13)	NMT1(17)	TRP1(9)	UGP1(3)	URA3(19)
G_19_12	59	FKS(7)	LEU2(13)	NMT1(17)	TRP1(9)	UGP1(3)	URA3(19)
G_20_6	59	FKS(7)	LEU2(13)	NMT1(17)	TRP1(9)	UGP1(3)	URA3(19)

G_19_8	59	FKS(7)	LEU2(13)	NMT1(17)	TRP1(9)	UGP1(3)	URA3(19)
G_18_6	59	FKS(7)	LEU2(13)	NMT1(17)	TRP1(9)	UGP1(3)	URA3(19)
G_20_2	59	FKS(7)	LEU2(13)	NMT1(17)	TRP1(9)	UGP1(3)	URA3(19)
G_18_2	178	FKS(7)	LEU2(16)	NMT1(9)	TRP1(32)	UGP1(50)	URA3(50)
G_19_13	8	FKS(1)	LEU2(2)	NMT1(2)	TRP1(1)	UGP1(2)	URA3(1)
G_18_3	8	FKS(1)	LEU2(2)	NMT1(2)	TRP1(1)	UGP1(2)	URA3(1)
G_19_11	43	FKS(10)	LEU2(5)	NMT1(19)	TRP1(19)	UGP1(1)	URA3(17)
G_19_2	3	FKS(5)	LEU2(7)	NMT1(8)	TRP1(7)	UGP1(3)	URA3(6)
G_19_19	123	FKS(7)	LEU2(16)	NMT1(46)	TRP1(13)	UGP1(1)	URA3(8)
G_19_1	18	FKS(7)	LEU2(4)	NMT1(3)	TRP1(4)	UGP1(1)	URA3(3)
G_18_10	26	FKS(7)	LEU2(4)	NMT1(3)	TRP1(4)	UGP1(1)	URA3(8)
G_21_1A	26	FKS(7)	LEU2(4)	NMT1(3)	TRP1(4)	UGP1(1)	URA3(8)
G_21_1B	26	FKS(7)	LEU2(4)	NMT1(3)	TRP1(4)	UGP1(1)	URA3(8)
G_20_13	15	FKS(8)	LEU2(5)	NMT1(3)	TRP1(5)	UGP1(1)	URA3(1)
G_20_3	83*	FKS(10)	LEU2(9)	NMT1(19)	TRP1(~5)	UGP1(1)	URA3(2)
G_20_7	83	FKS(10)	LEU2(9)	NMT1(19)	TRP1(5)	UGP1(1)	URA3(2)
G_21_2	83	FKS(10)	LEU2(9)	NMT1(19)	TRP1(5)	UGP1(1)	URA3(2)
G_21_4	83	FKS(10)	LEU2(9)	NMT1(19)	TRP1(5)	UGP1(1)	URA3(2)
G_19_17	83	FKS(10)	LEU2(9)	NMT1(19)	TRP1(5)	UGP1(1)	URA3(2)
G_18_5	83	FKS(10)	LEU2(9)	NMT1(19)	TRP1(5)	UGP1(1)	URA3(2)
G_20_12	83	FKS(10)	LEU2(9)	NMT1(19)	TRP1(5)	UGP1(1)	URA3(2)

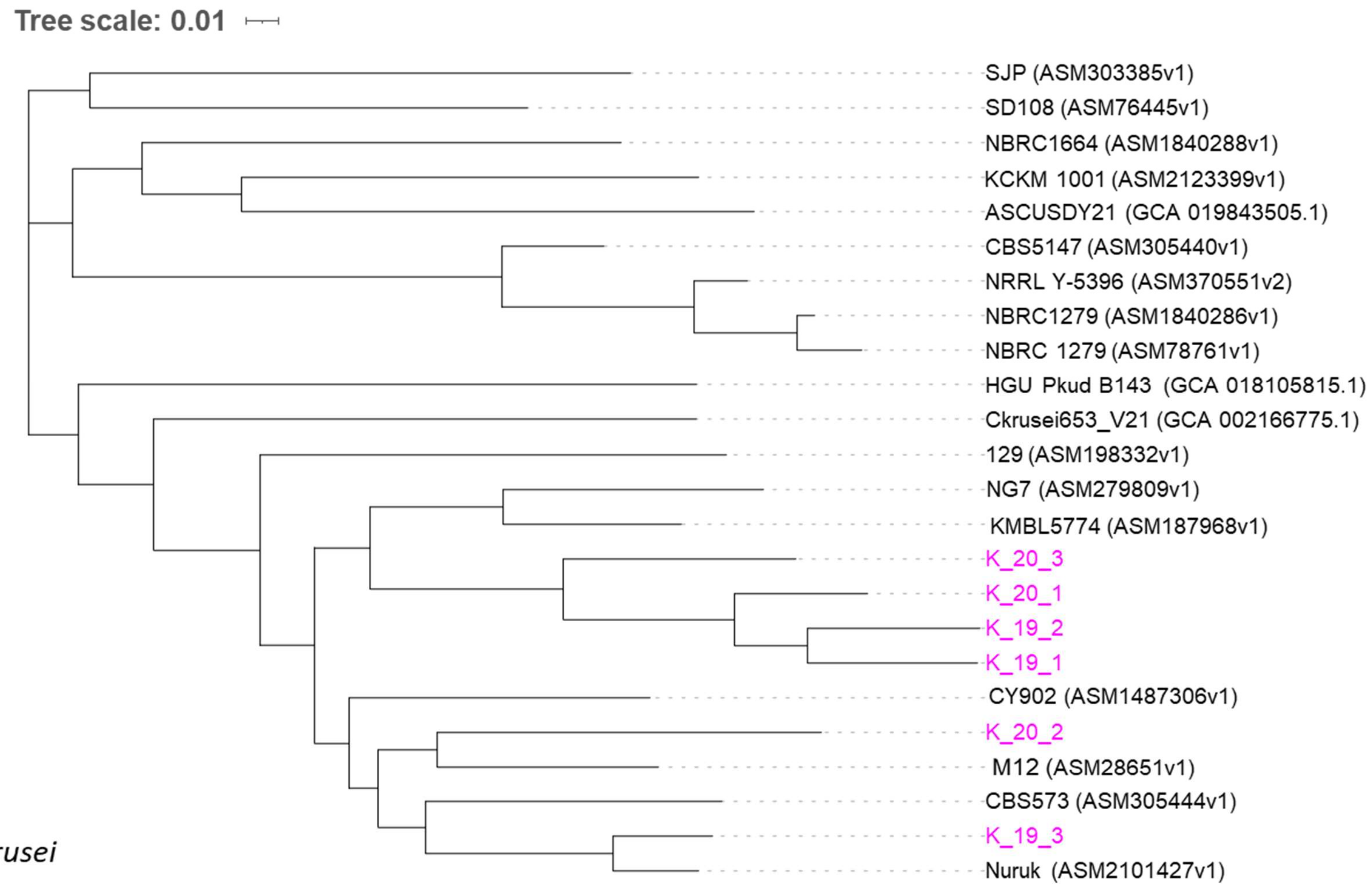

Candida krusei

Supplementary Figure 1. Phylogeny of the less common *Candida* species - *Candida dubliniensis*, *C. krusei*, and *C. auris*,. The trees were constructed from SKA (Split Kmer Analysis). GenBank accession numbers of assemblies used as comparators are written in parenthesis. Isolates sequenced in this study are have pink labels and have the prefix D_ (for *C. dubliniensis*), K_ (for *C. krusei)*, and A_ (for *C. auris)*. For *C. auris*, only the phylogeny of Clade 1 isolates were included. *C. auris* isolates Ca02, Ca03, Ca04 and Ca06 were isolated in Singapore, years 2012 – 2018.

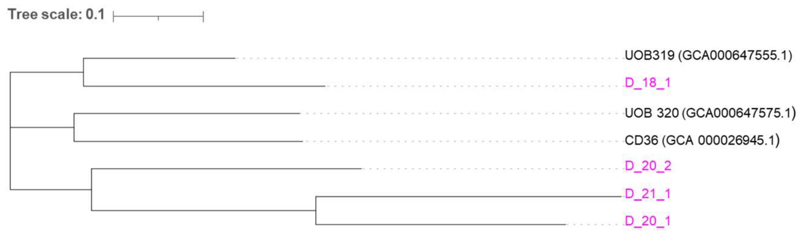

Candida dubliniensis

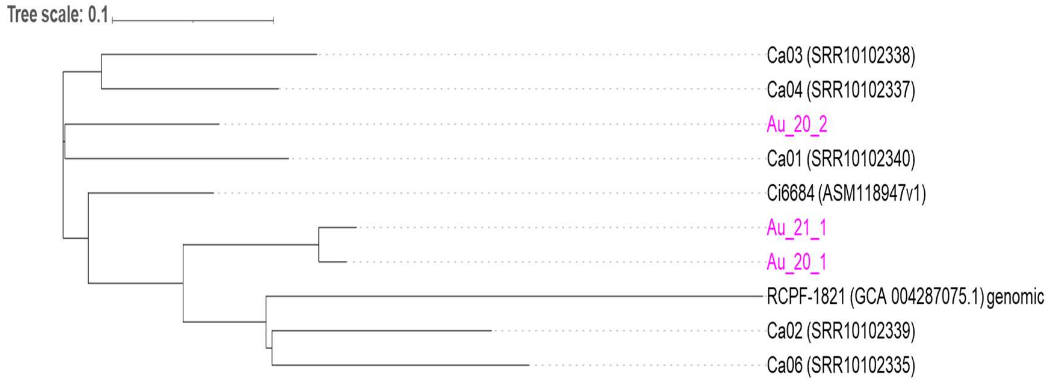

Candida auris
